# A dinosaurian facial deformity and the first occurrence of ameloblastoma in the fossil record

**DOI:** 10.1038/srep29271

**Published:** 2016-07-05

**Authors:** Mihai D. Dumbravă, Bruce M. Rothschild, David B. Weishampel, Zoltán Csiki-Sava, Răzvan A. Andrei, Katharine A. Acheson, Vlad A. Codrea

**Affiliations:** 1 Laboratory of Paleotheriology and Quaternary Geology, Faculty of Biology and Geology, BabeS-Bolyai University, 1, M. Kogălniceanu Str., 400084 Cluj-Napoca, Romania; 2 Department of Medicine, Northeast Ohio Medical University, Rootstown, 44505 Ohio, USA; 3 Center for Functional Anatomy and Evolution, Johns Hopkins School of Medicine, 1830 E. Monument St., Room 306 Baltimore, MD 21205, USA; 4 Faculty of Geology and Geophysics, University of Bucharest, 1, N. Bălcescu Blvd., 010041 Bucharest, Romania; 5 Ocean and Earth Science, National Oceanography Centre, University of Southampton, Waterfront Campus, European Way, Southampton, SO14 3ZH, UK

## Abstract

Despite documentation of various types of neoplastic pathologies encountered in the vertebrate fossil record, no ameloblastic tumours have been recognised so far. Ameloblastoma is a benign neoplasic tumour with a strong preponderance for the mandible. Here, we report for the first time the presence of an ameloblastoma neoplasm in the lower jaw of a specimen referred to the derived non-hadrosaurid hadrosauroid dinosaur *Telmatosaurus transsylvanicus* from the uppermost Cretaceous of the Ha
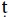
eg Basin in Romania. The location, external appearance and internal structure of the pathological outgrowth provide clear evidence for the diagnosis of ameloblastoma in *Telmatosaurus*. This report extends the range of pathologies encountered in hadrosauroid dinosaurs. In addition, recognition of an ameloblastoma neoplasm in a taxon lying close to the origin of ‘duck-billed’ hadrosaurid dinosaurs confirms the predisposition of this clade towards neoplasia pathologies already in its basal members.

Ancient diseases have been recognised for well over a century^1^ in dinosaurs^1^,^2^ and other pre-Cenozoic fossil vertebrate groups such as pterosaurs^3^, crurotarsans^4^, plesiosaurs^5^, mosasaurs^6^, ichthyosaurs^7^, turtles^8^, or synapsids^9^. Beside their intrinsic value in revealing a very particular facet of dinosaurian life and death – that is, the existence of certain diseases and their taxonomic and anatomic specificity, as well as of pathogenic agents responsible for them, studies into dinosaurian palaeopathologies have also contributed significantly to a more profound understanding of dinosaur behaviour.

The current range of documented dinosaurian pathologies is very wide^2^. Examples range from healed wounds from failed predator attacks^10^, intraspecific combat^11^ or daily routine activity leading to accidental injury and breakage^2^, to congenital and developmental defects^12^,^13^,^14^, cases of severe bacterial or fungal infection such as osteomyelitis^12^ following exogenous traumatic events, chronic diseases like osteoarthritis^15^, and neoplasms^16^,^17^.

Hadrosauroids were one of the most ecologically important, specious, and widespread clades of herbivorous dinosaurs during the ‘middle’ to Late Cretaceous. Hadrosauroids also rank among the most frequently cited examples of dinosaurs presenting pathological modifications^12^,^16^. This clade has been reported from the uppermost Cretaceous of western Romania based on remains collected for over a century from the Maastrichtian formations of the Ha
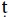
eg Basin and its surrounding areas^18^,^19^,^20^ (see Supplementary Figs 2 and 3). Despite early claims of pathological individuals from the Transylvanian record of dinosaurs (in accordance with the perceived ecologically strained nature of the local fauna^21^), subsequent research failed to substantiate such claims. Now, a hadrosauroid specimen from the Maastrichtian of the Ha
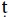
eg Basin provides the first evidence of a pathological condition (a neoplasm) previously unrecognized in the dinosaurian fossil record, and at the same time of a well-documented pathological modification in a Romanian dinosaur.

The specimen under consideration (Laboratory of Palaeontology Bucharest, Faculty of Geology and Geophysics, University of Bucharest [LPB (FGGUB) R.1305] was discovered in an outcrop of the Sînpetru Formation along the banks of the Sibişel River, in the central part of the Ha
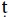
eg Basin (see Supplementary Figs S2 and S3). The specimen is represented by a pair of well-preserved, associated lower jaws with *in situ* dentition (Fig. 1A) belonging to a sub-adult individual of the hadrosauroid *Telmatosaurus transsylvanicus* (Nopcsa, 1900; see Supplementary Text S1). The elements are relatively small (right ramus: L-129 mm, W-34 mm, H-57 mm; left ramus: L-143 mm, W-58 mm, H-76 mm) only about half the size of the largest known *Telmatosaurus* individuals, including the lectotype skull [Natural History Museum, United Kingdom (NHMUK R.3386)]^18^,^19^. Although found in isolation, the dentaries were discovered close to their natural articular position, and not as part of a ‘bone-pocket’ assemblage, the most typical taphonomic mode described for this lithostratigraphic unit (see Supplementary Text S1). The less complete right ramus is broken posteriorly, level with the rostral edge of the coronoid process, while the better preserved left ramus preserves part of the coronoid process.

The labial surface of the left dentary has a smooth and shiny periosteum, except for an area covered by a thin crust of iron hydroxide deposits. It is also largely unworn and unabraded (Fig. 1A–D), only showing mainly transverse diagenetic fractures that fragmented the dentary into several parts that could be easily recombined to reveal the original morphology of the element. It is moderately convex dorsoventrally and roughly straight rostrodistally.

The pathology identified in LPB (FGGUB) R.1305, at a macroscopic examination, is represented by a prominent, rostroventrally slanting ridge-like bulge (exostosis) on the ventral two-thirds of the labial surface of the left dentary, close to its midline (Fig. 1A,C,D). The right counterpart of this dentary is similarly well-preserved, excepting some diagenetic fractures (see Supplementary Figs S5 and 3D Fig. S5). However, it does not show any external sign of a comparable deformation, neither on its labial face mirroring the bulge on the left side, nor in other parts of the element. As a result, the unilateral bony excrescence of the left dentary was considered pathological in nature and thus worthy of further investigations.

To investigate the precise nature of the lesion and to identify the pathogenic process responsible for producing it, detailed X-ray micro-CT scanning of the two jaw rami of LPB (FGGUB) R.1305 was employed (see Supplementary Fig. 1). Micro-CT examination of the left dentary segment showing the pathological bulge revealed a unique internal structure associated spatially with the external deformation visible by gross examination and has provided the necessary characteristics for a tentative diagnosis. Concurrent micro-CT investigation of the right ramus in the same area revealed no signs of any internal pathological features, thus confirming the unilateral presence of the observed lesion, localised exclusively around mid-length of the left dentary.

## Results

### Description of the pathology

**Figure 1:**
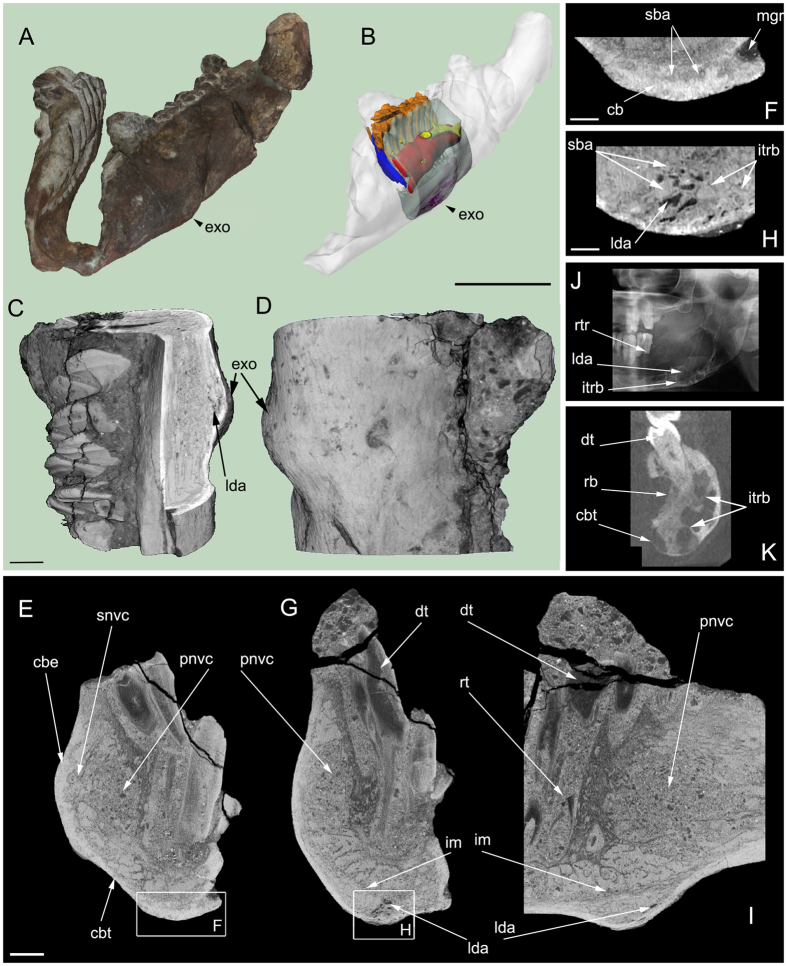
External morphology and internal structure of the pathological *Telmatosaurus transsylvanicus* dentary (LPB (FGGUB) R.1305). (**A**) Surface scans of the left and right rami positioned in anatomical articulation; (**B**) segmented internal structures of the left ramus exhibiting the pathology (red - primary neurovascular canal, yellow - secondary neurovascular pathways, orange - functional teeth, blue - replacement teeth, light blue - segmented dentary bone, purple - lytic density areas); (**C**) Three-dimensional micro-CT image with rectangular cutout in the area of the exostosis; (**D**) Three-dimensional micro-CT image of the investigated portion of the dentary showing the external appearance of the exostosis (**E**) transverse cross-section no. 260 micro-CT image in the coronal plane; (**F**) close-up detail of **E**. (**G**) Transverse cross-section no. 648 micro-CT image in the coronal plane; (**H**) close-up detail of **G**. (**I**) Transverse cross-section no. 775 micro-CT image in the sagittal plane; (**J**) comparative human panoramic radiograph of the left mandibular ramus exhibiting ameloblastoma (modified from^28^); (**K**) comparative human tomographic slice in the coronal plane of the left mandibular ramus exhibiting ameloblastoma (modified from^28^). Abbreviations: cb = cortical bone; cbe = cortical bone expansion; cbt = cortical bone thinning; dt = dentary tooth; exo = exostosis; im = internal margin; itrb = internal trabeculae; lda = lytic density areas; mgr = mandibular groove; pnvc = primary neurovascular canal; rb = resorbed bone; rt = resorbed tooth; rtr = resorbed tooth roots; sba = soap bubble appearance; snvc = secondary neurovascular pathways. Scale bars (**A**,**B)**: 50 mm. Scale bars (**C–I**): 5 mm. Scale for (**J**,**K**) not reported in the ref. 28.

The cortical bone surface at the site of the exostosis displays a locally lytic density (Fig. 1C,H,I, and Supplementary Figs S6f, S7e, S8f and 3D Fig. S4). Ventrally, the cortical bone shows moderate thinning (Fig. 1F) in the lower third of the left dentary, compared to the normal cortical thickness visible elsewhere in the specimen; at the ventral edge of the exostosis, however, there is a fair amount of expansion of the cortical bone (Fig. 1E). Between the modified cortical wall of the dentary labially, and the primary neurovascular canal and secondary neurovascular pathways (Fig. 1E,G,I) lingually, the internal structure of the pathological area consists of a pattern of large vacuolae separated by thin bony struts or internal trabeculae (Fig. 1J), reminiscent of a ‘soap bubble’ texture^22^,^23^,^24^,^25^,^26^,^27^ (Fig. 1F,H). At the dorsal apex of the exostosis, the cortical bone is expanded (Fig. 1E). The exostosis is separated from the normal trabecular bone by a thin layer of longitudinally expanded bony struts forming the internal margin of the lesion (Fig. 1G,I).

Although preliminary macroscopic assessment of the exostotic lesion on the left dentary suggested a callus resulting from healed fracture, micro-CT scanning failed to support such a cause. No displacement of the bone surface is visible on its exterior and, more importantly, no discernible internal disruption or fracture line affects the trabeculae and/or the teeth, anomalies that are generally associated with a fracture-induced lesion of the mandible as reported previously in the case of ceratopsian^11^ and derived hadrosauroid^12^ dinosaurs. Although some of the teeth in the pathological jaw section show partial resorption of roots (see Supplementary Figs S7d and S9g) and crowns (Fig. 1I), no obvious alteration is visible in the regular tooth replacement pattern characteristic of the typical hadrosauroid dental battery. There are no signs of tooth loss or misalignment, either.

### Differential diagnosis

The differential diagnosis for the pathology of the left dentary of *Telmatosaurus* LPB (FGGUB) R.1305 includes abscesses, odontogenic and non-odontogenic cysts, and neoplasia (see Supplementary Table 1). Intra-osseous odontogenic cysts include developmental cysts (follicular cysts, keratocysts and lateral periodontal cysts), inflammatory cysts (radicular cysts and paradental cysts), ameloblastoma, ameloblastic fibroma, odontogenic myxoma, primordial cyst and central odontogenic fibroma^23^,^24^,^26^. Inflammatory cysts derive from pulp necrosis induced by dental caries. Non-odontogenic cysts include traumatic bone cysts, solitary bone cysts, hemorrhagic cysts, unicameral bone cysts and traumatic bone cysts. Non-odontogenic neoplasia includes metastatic cancer, multiple myeloma/plasma cell tumour lymphoma, leukemia, hemangioma, neurofibroma, and schwannoma.

The localisation, external appearance and topology, and especially the internal osseous structure corresponding to the exostotic bulge, are consistent with a diagnosis of ameloblastoma, a benign odontogenic neoplasia^28^. Ameloblastomas are described as expansile multiloculated cystic or mixed cystic and solid areas with cortical thinning (Fig. 1K), and may be associated with tooth root resorption (Fig. 1J)^29^,^30^. Internal septations produce a ‘honeycomb’ or ‘soap bubble’ appearance^22^,^24^,^31^. They represent about 10% of odontogenic tumours in humans, and are usually characteristic to young adults^32^.

The identified pathologic structure resembles a dental abscess, a pathology reported previously to occur as early as the Permian in the captorhinid *Labidosaurus*^9^, and which is also recognised in Late Cretaceous hadrosauroids^12^. Development of a marked labial bony overgrowth on the dentary is a feature shared between the pathology of the Romanian hadrosauroid dentary and that of a Canadian latest Cretaceous hadrosaur first documented in detail^31^ to represent a case of dental abscess. However, the irregular borders and septate nature of the inner structure presented by the pathologic left dentary of LPB (FGGUB) R.1305 are not compatible with that diagnosis; moreover, tooth loss that is usually associated with dental abscesses is not noted in the Romanian dentary.

Osteomyelitis, a disease often spread from traumatised and/or infected teeth more deeply into the dentary, has a lengthy fossil record, recognised in the Early Permian captorhinid *Labidosaurus*^9^ as well as in the Early Jurassic basal theropod *Sinosaurus*^32^ and in Late Cretaceous ceratopsians^11^. Acute suppurative osteomyelitis, while osteolytic, is non-expansile, in contrast to chronic osteomyelitis in which osteolytic expansion is accompanied by bony sequestra^28^,^33^, whereas sclerosing osteomyelitis is characterised by diffuse sclerotic expansion; neither of these features are present in the Romanian dentary. Osteomyelitis is also often associated with development of draining sinuses, the presence of which cannot be documented in LPB (FGGUB) R.1305.

The pathological condition recognised in the *Telmatosaurus* dentary is distinguished from ameloblastic fibroma by lack of the internal calcifications characteristic of the latter, and from ameloblastic carcinoma by absence of bone destruction^31^,^33^,^34^. The pathology in the dentary is clearly distinguished from dentrinous tumours, odontoma, enameloma, periapical cement dysplasia, ossifying fibroma, and renal osteodystrophy, which have increased, rather than decreased density on X-ray examination^28^,^33^.

Osteosarcomatous lesions are ill-defined and tend to be osteoblastic, unlike the pathology in LPB (FGGUB) R.1305. While there are similarities of this pathology to giant cell reparative granuloma^28^,^33^,^35^, the latter do not have internal septations. Furthermore, the pathology in *Telmatosaurus* does not have the scalloped appearance of tuberculosis, the topographic appearance of eosinophilic granuloma, the ill-defined surface of odontogenic myxoma, fibrosarcoma, and intraosseous hematoma, the rough surface of metastatic malignancies, the internal trabecular pattern (Fig. 1J) of hemangioma and osteosarcoma, or the ground glass appearance of fibrous dysplasia, ossifying fibroma, Paget’s disease and hyperparathyroidism^28^,^33^,^36^. Root resorption in ameloblastoma clearly distinguishes it from the preserved, but floating teeth of eosinophilic granuloma. The specimen lacks the increased tooth separation of globulomaxillary cysts. Follicular cysts contain unerupted teeth with bone remodelling rather than expansion^33^. The pathology in LPB (FGGUB) R.1305 is also clearly distinguished from the lingual cortical defect referred to as a Stafne cyst, and from the spheroid expansile lesions of multiple myeloma which penetrate rather than expand bone^26^,^27^,^28^.

In conclusion, although the pathological condition reported here in the dentary of the hadrosauroid *Telmatosaurus transsylvanicus* is by no means unequivocally pathognomonic, its characteristics nevertheless suggest identification as an ameloblastoma. The presence of this benign neoplasic modification is reported here for the first time in the dinosaurian fossil record, as well as in the fossil record overall. The occurrence of ameloblastoma is a well-known phenomenon in humans^37^ and other mammals^38^, yet has only recently been identified among reptiles in the living wild black rat snake^39^. A related disorder, adenoameloblastoma, was noted in the Indian rock python^40^.

## Discussion

When first suggesting the insular nature of the latest Cretaceous Ha
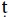
eg fauna, Nopcsa^21^ noted several palaeobiological features in support of his views, including what he perceived as the common presence of pathological individuals. He considered this condition a reasonable consequence of the ecologically impoverished and stressed environment inhabited by this fauna. Identification of pathological individuals was, however, largely unsuccessful subsequently; there is, as yet, no thoroughly described and diagnosed case of dinosaurian pathology reported from the Transylvanian fossil record, although possible cases of dinosaur neoplasm pathologies have been noted previously^41^,^42^,^43^.

Now, recognition of ameloblastoma in a *Telmatosaurus* dentary discovered from the same area represents the fourth reported, and best documented, case of pathological modification identified in Transylvanian dinosaurs, all of which appear to represent neoplasic diseases^41^,^42^,^43^. Our report might lend some support to Nopcsa’s^21^ view about high incidence of pathologies in the Transylvanian dinosaur fauna, although these cases remain still scarce.

Even more remarkable is the taxonomic preference and nature of these Transylvanian pathologies. All reported cases belong to hadrosauroids, and all appear to represent tumoural modifications of neoplasic origin. Although neoplasia pathologies have been reported from as early as the Late Jurassic^43^, tumoural modifications appear to be relatively rare in the dinosaurian fossil record^2^,^44^. While the neoplasia incidence rates in dinosaurs appear to be statistically comparable with that known in Recent animals^45^, neoplasic modifications demonstrate a high level of taxonomic selectivity for hadrosauroids. The study of Rothschild *et al*.^16^ showed that neoplasia pathologies (including malignant forms) were restricted to hadrosauroids in their extensive sample covering all major dinosaur clades; the first case of dinosaurian neoplasia occurring outside the Hadrosauroidea was reported only recently, in the form of haemangioma and osteoma identified in a Late Cretaceous titanosaur from Brazil^17^. Haemangiomas have been identified in several hadrosauroids^16^, including the derived non-hadrosaurid hadrosauroids *Gilmoreosaurus* and *Bactrosaurus*, as well as the saurolophines *Edmontosaurus* and *Brachylophosaurus*. Meanwhile, cases of benign neoplasia such as desmoplastic fibroma and osteoblastoma, as well as of metastatic cancer were found only in *Edmontosaurus*^16^.

This report of an ameloblastic *Telmatosaurus* dentary not only expands the spectrum of neoplasia reported in dinosaurs (and more specifically in hadrosauroids), but it provides further support for an apparent predisposition of hadrosauroids for neoplasia pathologies^16^. Since different types of neoplasia are reported to occur already in diverse derived non-hadrosaurid hadrosauroids (*Telmatosaurus, Bactrosaurus* and *Gilmoreosaurus*), it appears that this particular predisposition had already arisen in the ancestors of the true hadrosaurs.

**Figure 2:**
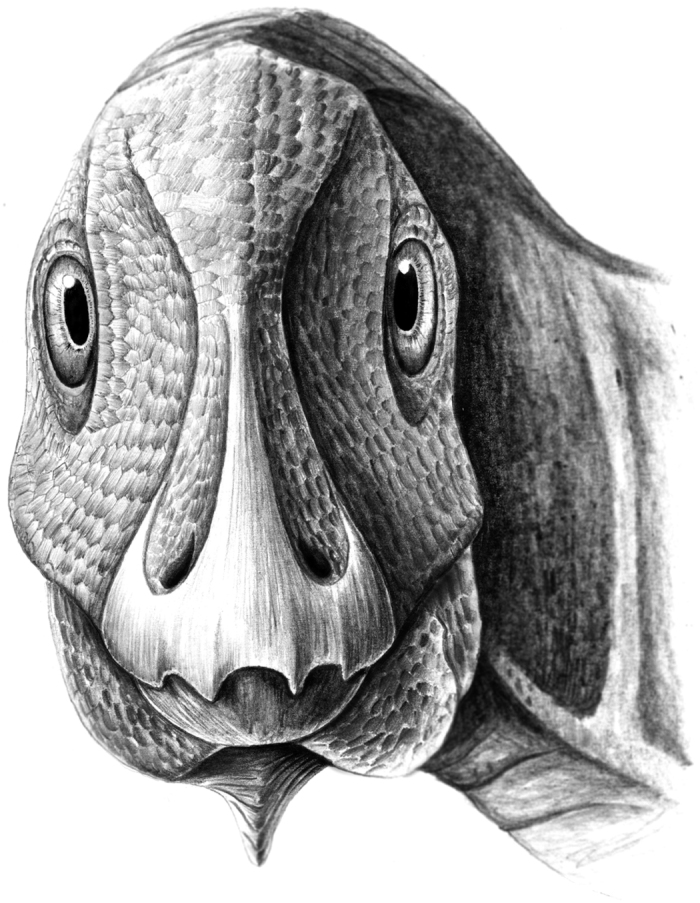
Artistic reconstruction of the pathological individual of the hadrosauroid *Telmatosaurus transsylvanicus* (LPB [FGGUB] R.1305) in rostral view, showing the probable life appearance of the mandibular deformity caused by ameloblastoma. (Reconstruction by M.D. D.).

Due to the limited nature of the fossil material referable to individual LPB (FGGUB) R.1305, no clear evidence for a cause of death is discernible in this subadult hadrosauroid specimen. Nevertheless, even if ameloblastoma itself is not lethal, its uncontrolled development and potential spread to other areas of the muzzle can eventually led to more severe abnormalities of the buccal and narial areas. Such abnormalities could become severely debilitating, and might have finally contributed to the demise of this individual. Furthermore, the *in vivo* appearance of the exostosis (Fig. 2) would have set this particular individual apart from other herd members, possibly leaving it vulnerable to predator attack. Such a predisposition of predators to select and attack individuals bearing malformations has been noted before in the case of Antarctic penguin chicks preyed upon by Skua (*Stercorarius* sp.)^46^. Another example of selective predation upon malformed individuals was described by Goodman and Johnson^47^, where individuals of the Pacific chorus frog (*Pseudacris regilla*) which showed parasite-induced limb malformations underwent alterations in predator avoidance behaviour; such alterations, in turn, allow predators to approach closer than normal their crippled frog prey, thereby severely reducing its chances of escape. Also, there is a considerable body of evidence correlating bilateral symmetry and sexual selection across several major taxa and a variety of species^48^. Whether such a selective intra-specific behaviour would further imply that malformed individuals such as the sub-adult *Telmatosaurus* LPB (FGGUB) R.1305 reported here were also marginalized within their group, and therefore made more exposed to predators, is not known but can be certainly conjectured.

## Methods

### Micro-CT Scanning

Given the rarity of pathological specimens in the dinosaurian material from the Ha
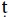
eg Basin, the non-destructive method of micro-CT scanning was chosen to investigate the internal nature of the identified surface pathology. Both left (see Supplementary Figs 1 and 4) and right (see Supplementary Fig. 5) rami were scanned with a SCANCO Medical μCT 100 machine at SCANCO Medical AG headquarters in Zürich, Switzerland. The scanning was supervised by Dr. Răzvan Andrei and conducted by Dr. Lisa Falco. Due to the large dimensions of the specimens and the prohibitive size of the resulting dataset, only the region of interest (L-32.22 mm, W-28.28 mm, H-55.38 mm) exhibiting the pathology was scanned on the pathological left dentary (see Supplementary Fig. 1e). To facilitate comparison and help determine the type of pathological change encountered, the corresponding areas of both dentaries were scanned using the same equipment parameters.

Scanning was made with a reflection micro focus X-ray source having a focal spot size of 12 μ, powered by a current of 90 kVp at an intensity of 150 mAs providing a spatial resolution of 4 μm per voxel. Beam hardening was accomplished by the use of a 0.5 mm copper filter. The specimens were rotated by 0.25° between successive projections. At each 0.25° increment, three exposures were made with a flat panel X-ray detector and the resulting images were digitally stacked to enhance the signal to noise ratio and suppress random digital noise. A total of 720 images were generated for a complete 180° rotation. The images were subsequently reconstructed using a back-projection algorithm to obtain the full 360° view. The 3D volume thus obtained was sliced axially in the region of the exostosis, resulting in 1249 individual slices for the right ramus and 1305 slices for the left. Each 0.025 mm thick slice was saved as a grayscale, 16-bit 1200 dpi DICOM and TIF file with a resolution of 2048 × 2048 pixels (see Supplementary Fig. 1c). The TIF files were saved with an LZW compression algorithm. The DICOM dataset is 4.8 Gb for the right ramus and 3.3 Gb for the left one. The TIF files were imported into Adobe Photoshop CS3 and processed globally for contrast improvement and saved as 8-bit JPEG files to reduce the size of the dataset (see Supplementary Fig. 1d). The two datasets were imported individually into FEI Amira 5.3.3 (Visualization Sciences Group) for viewing, analysis and segmentation (see Supplementary Fig. 1c). All micro-CT datasets were analysed on a Lenovo ThinkPad W520 64-bit mobile workstation with 32 Gb of RAM and an nVidia Quadro 2000 M graphics card with 2 Gb of RAM running Windows 7 Professional Edition. Structures of interest such as the teeth, external mandibular artery, primary neuro-vascular canal and cortical bone surface were highlighted and labelled (see Supplementary Fig. 1f) using the manual segmentation tools in Amira. Each structure was then saved in STL format and aligned relative to the other structures. The datasets are available for download upon request, from the Morphosource website (http://morphosource.org/index.php/Detail/SpecimenDetail/Show/specimen_id/2089).

### 3D Surface Scanning

To integrate the micro-CT data into the complete element, both rami were surface scanned by one of the authors (M. D. D.) using the 3D photogrammetry software Photoscan PRO 1.1.6 (Agisoft LLC).

The 360° photographic coverage was repeated from 4 different orientations to record the complete surface. The individual surface scans (see Supplementary Fig. 1g) were subsequently aligned in Photoscan to obtain a closed mesh. The 3D surface (see Supplementary Fig. 1h) was saved as an STL format.

Both the segmented internal structures and the 3D surface scan were subsequently imported into Rhinoceros 5.0 (Robert McNeill and Associates 2015, Release 2015-8-10) where the segmented structures were aligned within the 3D surface scan of the mandible and assigned levels of opacity and colour to differentiate between the various structures and emphasise their spatial and dimensional relationships (see Supplementary Fig. 1i). The aligned structures were then combined into 3D PDF files using Adobe Acrobat 10 and the 3D PDF plugin (Tech Soft 3D, Inc. DBA tetra 4D). The micro-CT datasets were animated in Photoshop CS3 to produce Supplementary Movies 1 and 2. The two DICOM datasets were saved as 3D. tif images for online repository storage and access (see Acknowledgements).

### Photography

The specimen was also photographed in standard anatomical orientations with a Canon EOS 500D DSLR camera equipped with a Nikon DG 60 mm EDX lens and a macro ring light. Prior to photographing, the specimens were lightly coated with ammonium chloride (NH_3_Cl) to improve contrast. All photographs were processed globally in Adobe Photoshop CS3 to improve contrast and suppress camera noise (see Supplementary Fig. 1b). The entire workflow (see Supplementary Fig. 1) was identical for both mandibles.

## Additional Information

**How to cite this article**: Dumbravă, M. D. *et al*. A dinosaurian facial deformity and the first occurrence of ameloblastoma in the fossil record. *Sci. Rep.* **6**, 29271; doi: 10.1038/srep29271 (2016).

## Supplementary Material

Supplementary Information

Supplementary Movie S1

Supplementary Movie S2
