# Supplementary material for: A dinosaurian facial deformity and the first occurrence of ameloblastoma in the fossil record
Source: Sci Rep. 2016 Jul 5;6:29271. doi: 10.1038/srep29271 (PMC4932493; doi:10.1038/srep29271)
Supplement: Supplementary Information [file srep29271-s1.pdf]

A dinosaurian facial deformity and the first occurrence of ameloblastoma in the fossil record.

Mihai D. Dumbravă[1*], Bruce M. Rothschild[2], David B. Weishampel[3], Zoltán Csiki-Sava[4], Răzvan A. Andrei[4], Katharine A. Acheson[5], Vlad A. Codrea[1]

[1]*Laboratory of Paleotheriology and Quaternary Geology, Faculty of Biology and Geology, Babeș-Bolyai University, 1, M. Kogălniceanu Str., 400084 Cluj-Napoca, Romania;* lilliensternus@gmail.com, codrea_vlad@yahoo.fr; [2]*Department of Medicine, Northeast Ohio Medical University, Rootstown, 44505 Ohio, USA,* spondylair@gmail.com; [3]*Center for Functional Anatomy and Evolution, Johns Hopkins School of Medicine, 1830 E. Monument St., Room 306 Baltimore, MD 21205 USA,* dweisham@jhmi.edu; [4]*Faculty of Geology and Geophysics, University of Bucharest, 1, N. Bălcescu Blvd., 010041 Bucharest, Romania,* zoltan.csiki@g.unibuc.ro, razvan.a.andrei@gmail.com; [5]*Ocean and Earth Science, National Oceanography Centre, University of Southampton Waterfront Campus, European Way, Southampton, SO14 3ZH, UK,* k.acheson@soton.ac.uk.

SUPPLEMENTARY INFORMATION

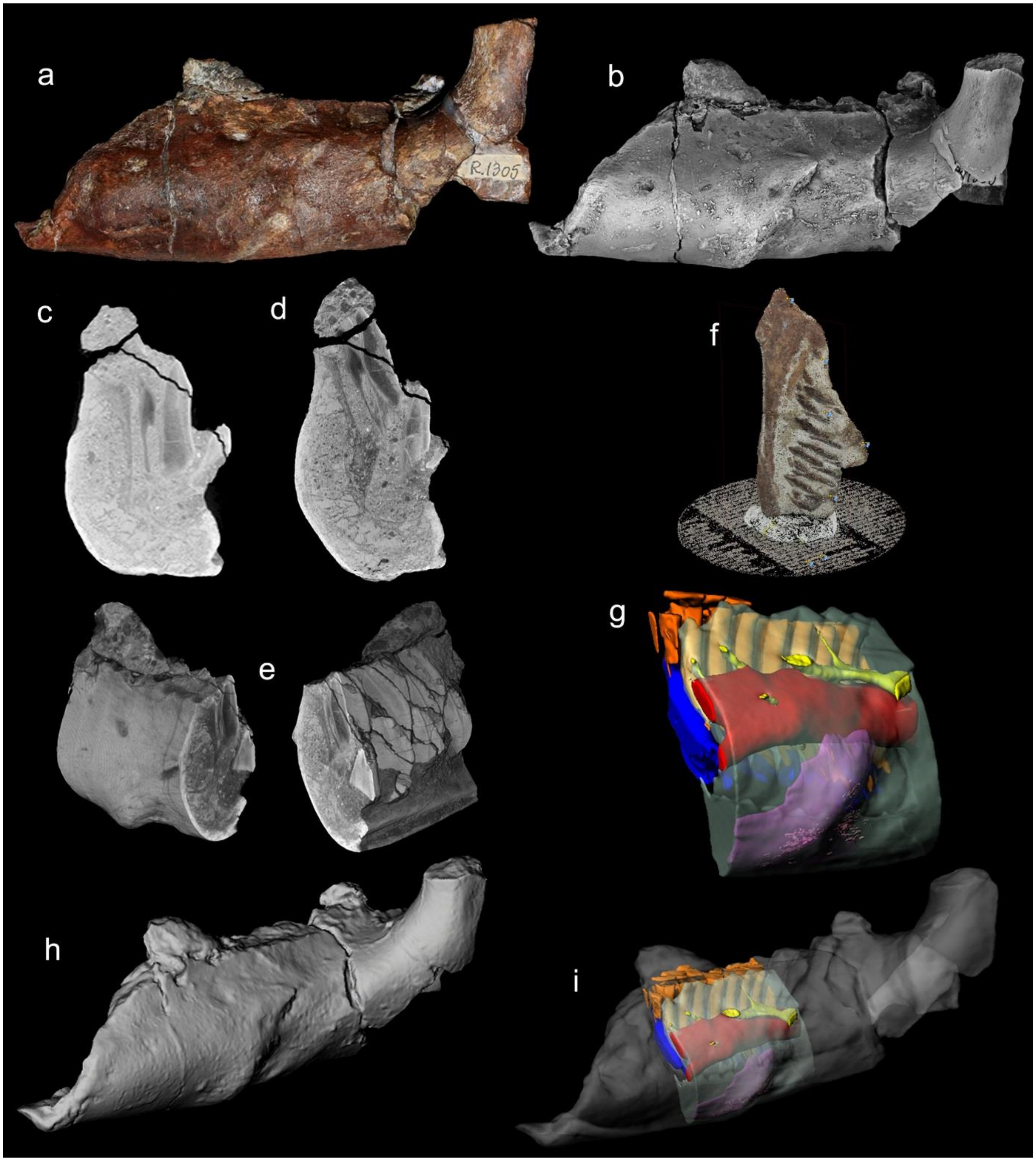

Supplementary Figure 1. Graphical depiction of the workflow. (**a**) Photograph of specimen in lateral view. (**b**) ammonium chloride coated photograph. (**c**) raw DICOM slice. (**d**) Processed TIFF slice. (**e**) Stacked slices comprising the 3D volume. (**f**) Photoscan PRO point cloud with texture. (**g**) Segmentation of internal structures. (**h**) Closed 3D surface scan. (**i**) composite 3D model.

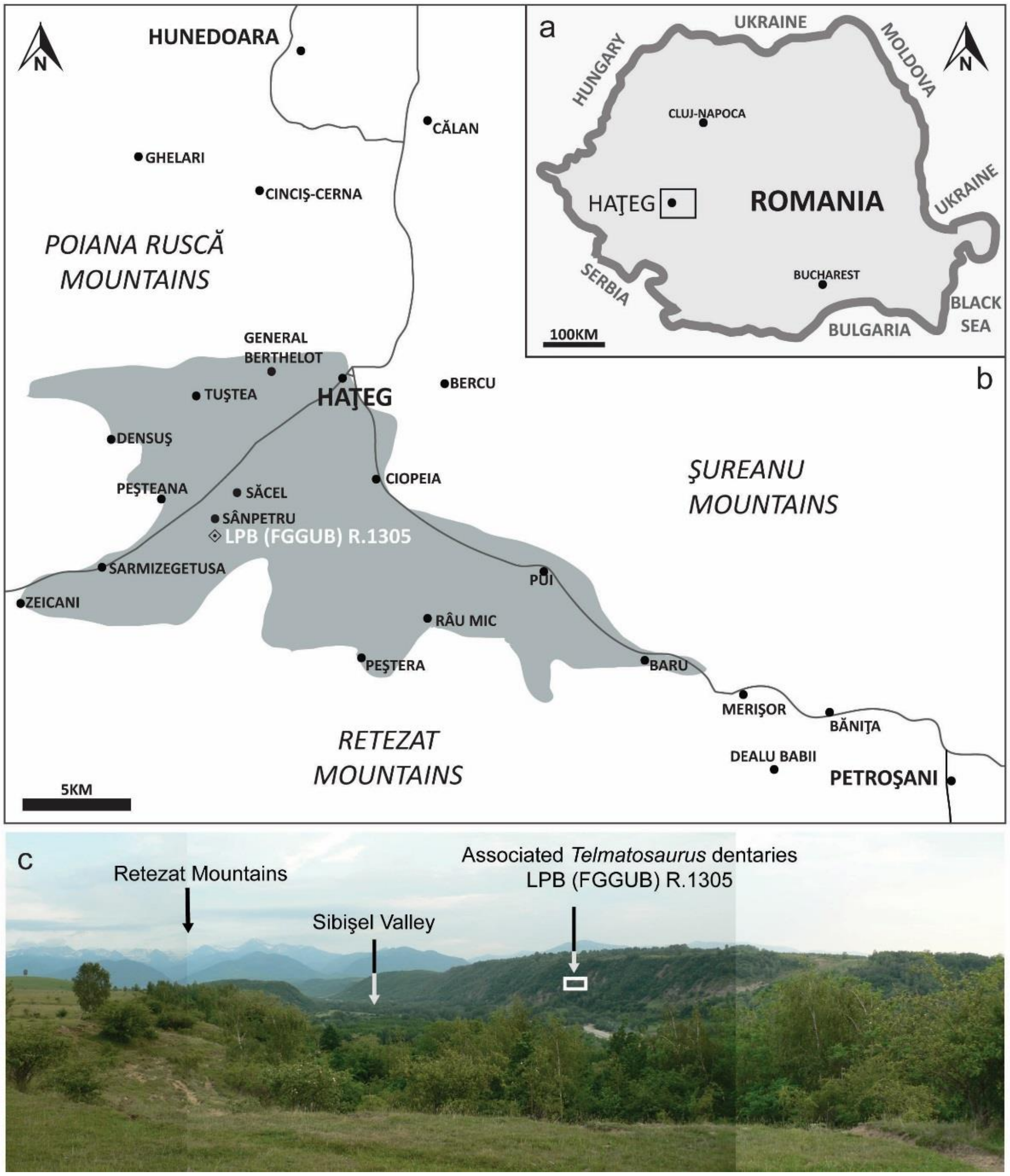

Supplementary Figure 2. Geographical location of the *Telmatosaurus transsylvanicus* (LPB [FGGUB] R.1305) dentaries. (**a**) location of the Haţeg Basin within Romania (by K. A. A in Adobe Illustrator CS6 version 16.0..5 http://www.adobe.com/support/downloads/detail.jsp?ftpID=5777). (**b**) extent (solid light grey), main vertebrate bearing localities and major population centers in and around the Haţeg Basin (by K. A. A. in Adobe Illustrator CS6 version 16.0..5 http://www.adobe.com/support/downloads/detail.jsp?ftpID=5777). (**c**) panoramic view of the Sibișel Valley looking southwards and towards the left slope (photographs by Z. Cs.-S.).

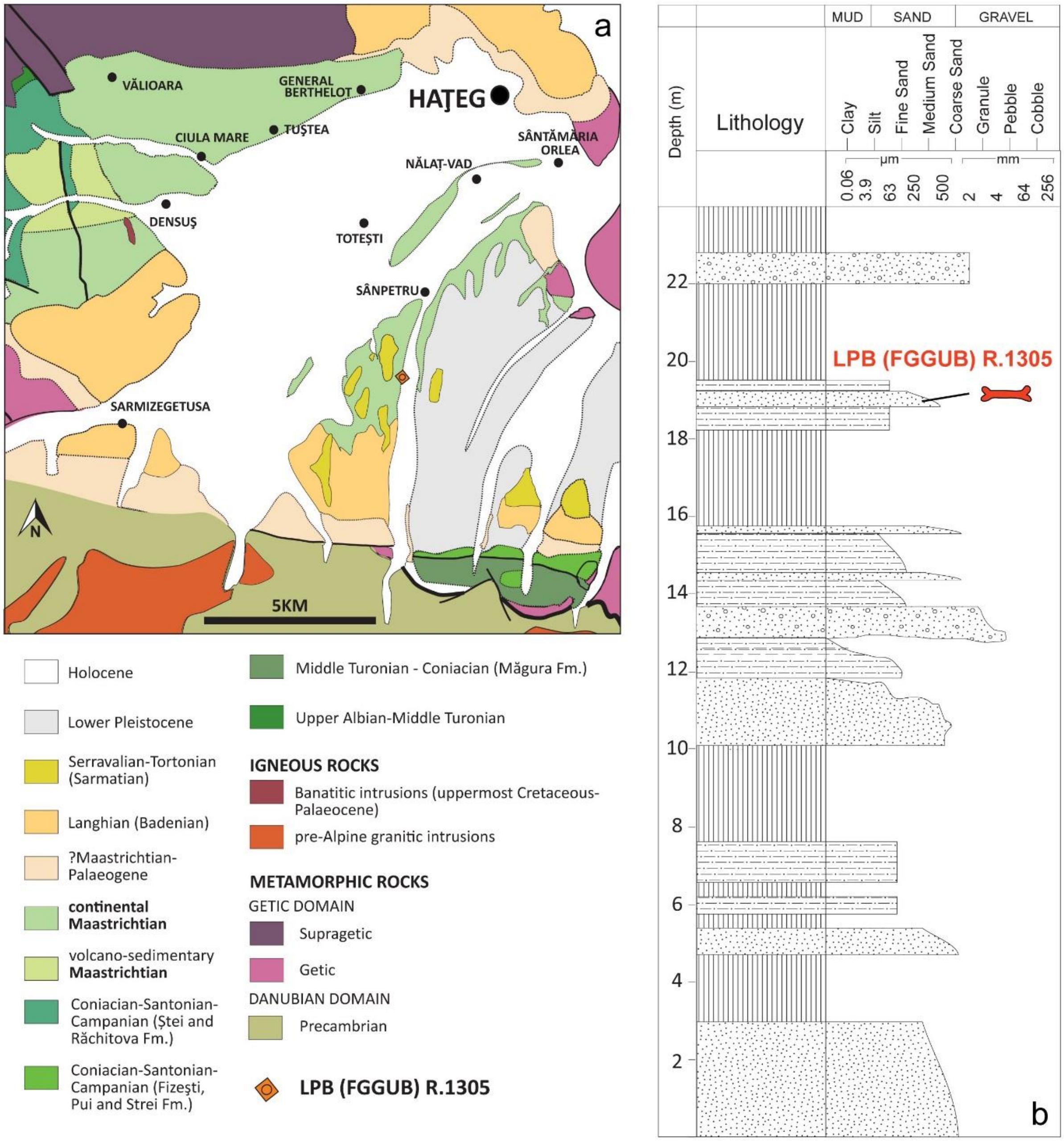

Supplementary Figure 3. Geology of the Haţeg basin and stratigraphy of the discovery site. (**a**) Geological map of the southern part of the Haţeg Basin (by Z. Cs.-S. in CorelDRAW Graphics Suite X7 http://www.coreldraw.com/us/product/graphic-design-software/). Scale bar is 5Km. (**b**) Lithostratigraphic log in the middle part of the Sibişel Valley section, Sînpetru Formation, showing the stratigraphic position of LPB (FGGUB) R.1305 (by K. A. A. in Adobe Illustrator CS6 version 16.0..5 http://www.adobe.com/support/downloads/detail.jsp?ftpID=5777).

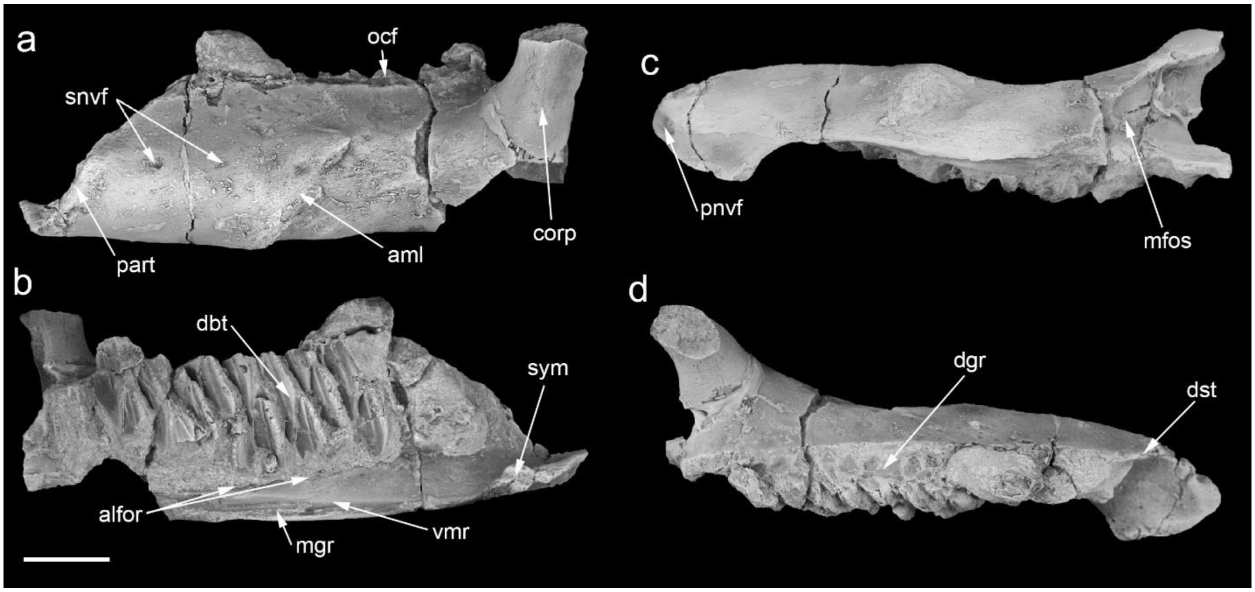

Supplementary Figure 4. Dentary of *Telmatosaurus transsylvanicus* (LPB [FGGUB] R.1305), pathologic left ramus. (**a**) lateral view. (**b**) medial view. (**c**) ventral view. (**d**) occlusal view. Abbreviations: alfor, alveolar foramina; aml, ameloblastoma; corp, coronoid process; dbt, dental battery; dgr, dental groove; dst, diastema; mfos, Meckelian fossa; mgr, mandibular groove; nfor, neurovascular foramina; ocf, occlusal facet; part, predentary articulation; pnvf, primary neurovascular foramen; snvf, secondary neurovascular foramina; sym, symphysis; vmr, ventromedial ridge. Scale bar is 20mm.

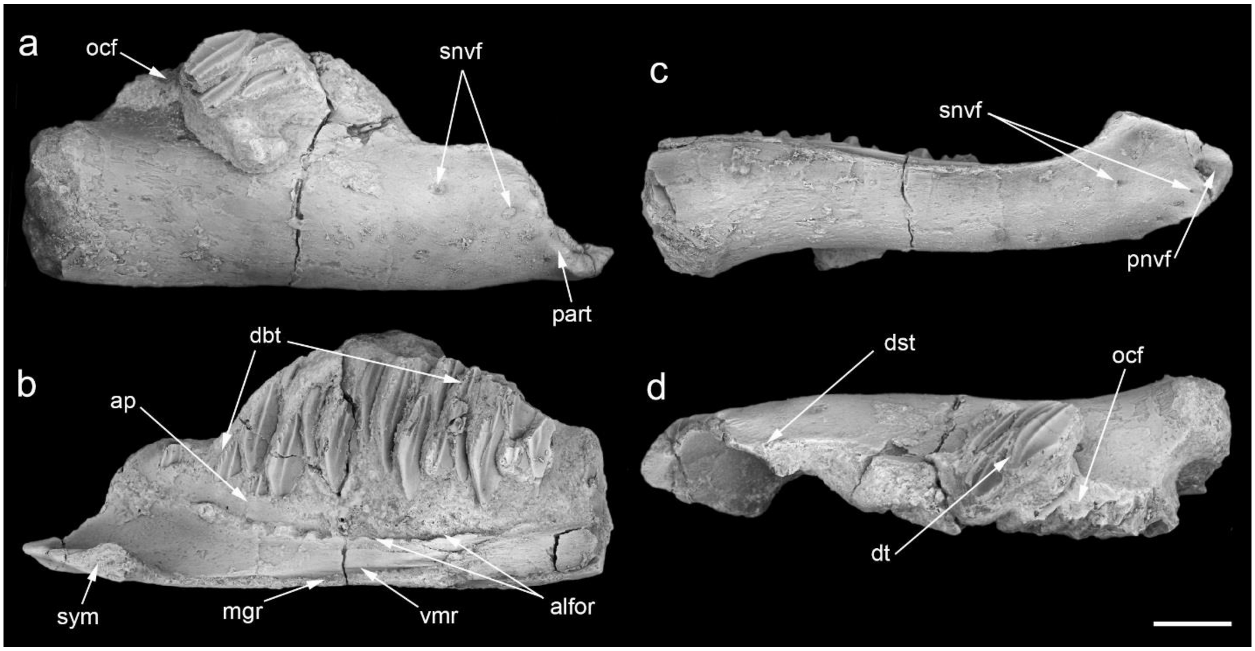

Supplementary Figure 5. Dentary of *Telmatosaurus transsylvanicus* (LPB [FGGUB] R.1305), normal right ramus. (**a**) lateral view. (**b**) medial view. (**c**) ventral view. (**d**) occlusal view. Abbreviations: ap, alveolar parapet; alfor, alveolar foramina; dbt, dental battery; dt, displaced teeth; mgr, mandibular groove; ocf, occlusal facet; part, predentary articulation; pnvf, primary neurovascular foramen; snvf, secondary neurovascular foramina; sym., symphysis; vmr, ventromedial ridge. Scale bar is 20mm.

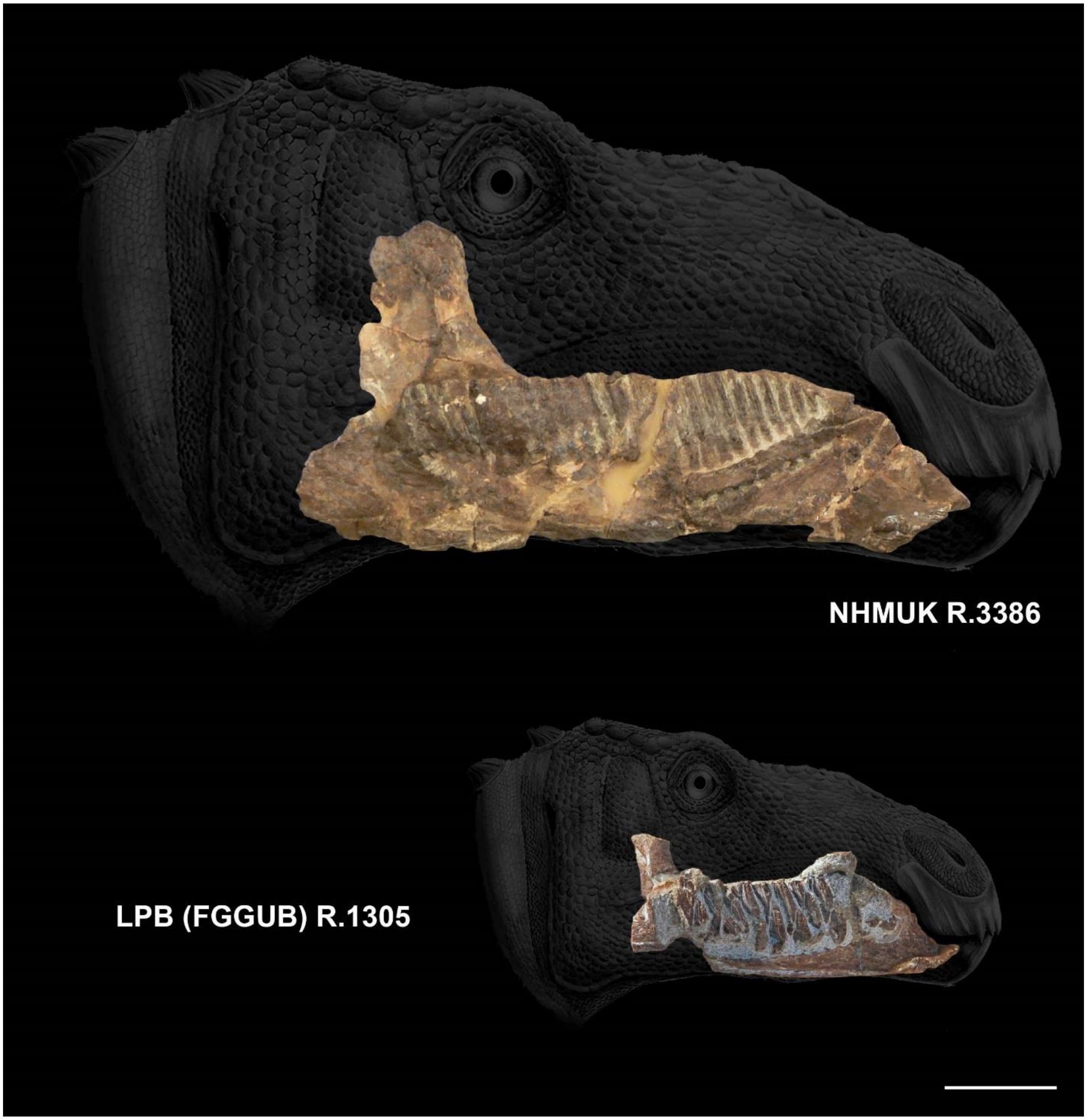

Supplementary Figure 6. Comparison between the left lectotype dentary of *Telmatosaurus transsylvanicus* (NHMUK R.3386; photo Z. Cs.-S.) and the referred pathological specimen (LPB [FGGUB] R.1305), in medial views. Tentative reconstruction of relative cranial size scaled from dentary dimensions between adult (based on the lectotype) and sub-adult (based on the referred pathological specimen) individuals. Scale bar is 50mm.

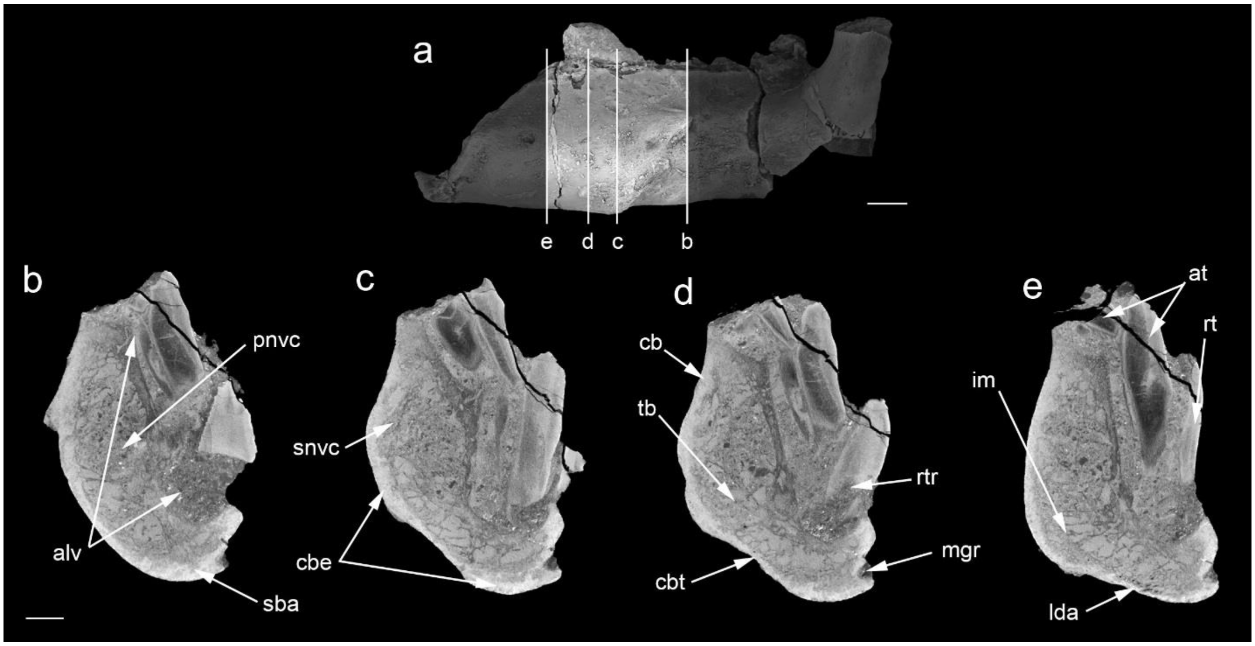

Supplementary Figure 7. Dentary of *Telmatosaurus transsylvanicus* (LPB [FGGUB] R.1305), pathologic left ramus, diagnostic micro-tomographic slices in the axial plane. (**a**) general view illustrating the scan area and the range of slices selected. (**b**) micro–CT image of axial slice no. 0000. (**c**) micro–CT image of axial slice nr. 0218. (**d**) micro–CT image of axial slice no. 0280. (**e**) micro–CT image of axial slice no. 0409. Abbreviations: alfor, alveolar foramina; alv, dentary alveoli; at, active teeth; cb, cortical bone; cbe, cortical bone expansion; cbt, cortical bone thinning; lda, lytic density areas; mgr, mandibular groove; pnvc, primary neurovascular canal; rt, replacement tooth; rtr, resorbed tooth roots; sba, soap bubble appearance; snvc, secondary neurovascular canal; tb, trabecular bone; im, internal margin. Scale bar is 10mm for a and 5mm for b, c, d, e.

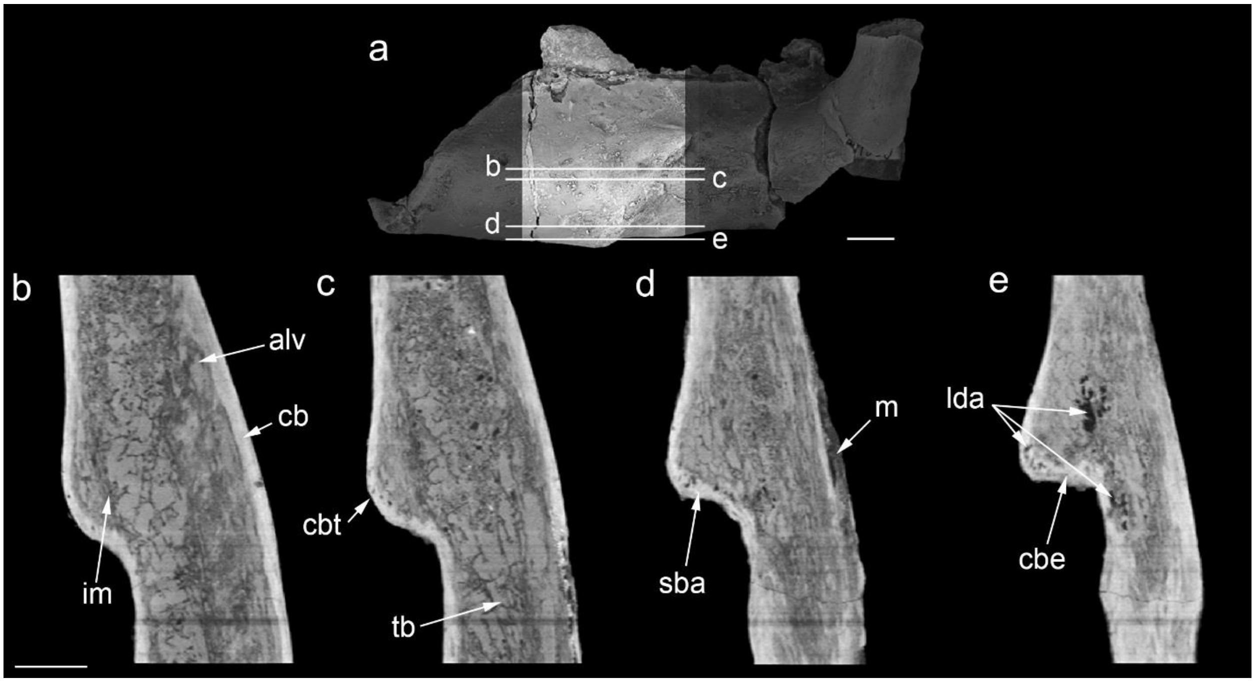

Supplementary Figure 8. Dentary of *Telmatosaurus transsylvanicus* (LPB [FGGUB] R.1305), pathologic left ramus diagnostic micro-tomographic slices in the coronal plane. (**a**) general view illustrating the scan area and the range of slices selected. (**b**) micro–CT image of coronal slice no.0269. (**c**) micro–CT image of coronal slice no. 0277. (**d**) micro–CT image of coronal slice no. 0294. (**e**) micro–CT image of coronal slice no. 0301. Abbreviations: alv, dentary alveoli; cb, cortical bone; cbe, cortical bone expansion; cbt, cortical bone thinning; lda, lytic density areas; m, matrix; sba, soap-bubble appearance; tb, trabecular bone ; im, internal margin. Scale bar is 10mm for a and 5mm for b, c, d, e.

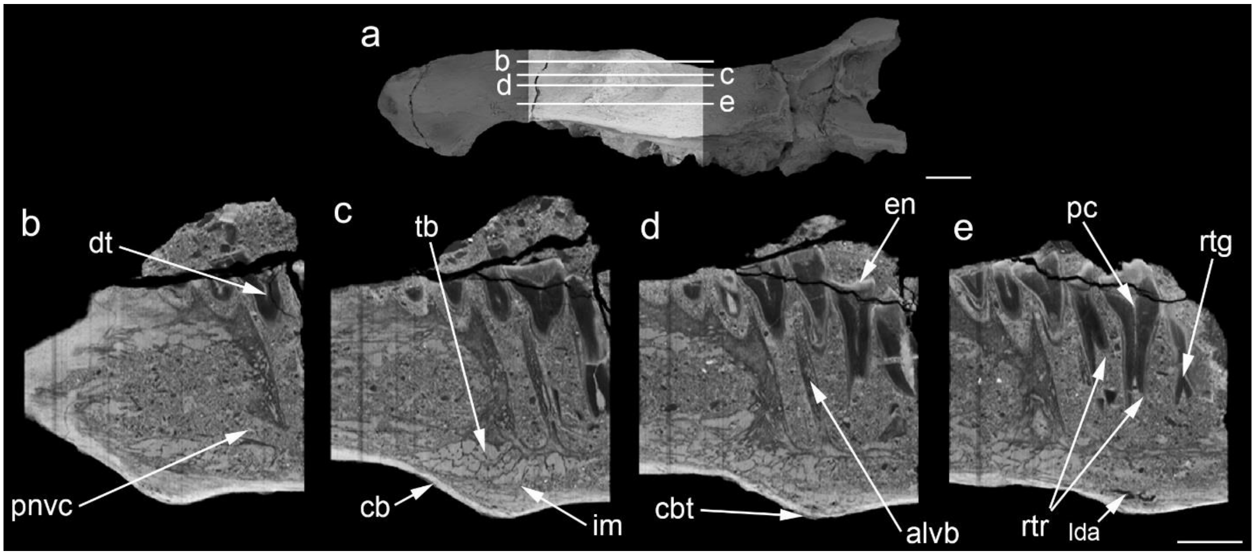

Supplementary Figure 9. Dentary of *Telmatosaurus transsylvanicus* (LPB [FGGUB) R.1305), pathologic left ramus, diagnostic micro-tomographic slices in the sagittal plane. (**a**) general view illustrating the scan area and the range of slices selected. (**b**) micro–CT image of sagittal slice no. 0144. (**c**) micro–CT image of sagittal slice no. 0166. (**d**) micro–CT image of sagittal slice no. 0179. (**e**) micro–CT image of sagittal slice no. 0193. Abbreviations: alv, dentary alveoli; alvb, alveolar bone; cb, cortical bone; cbt, cortical bone thinning; dt, dentary tooth; en, enamel; im, internal margin; lda, lytic density areas; pc, pulp cavity; pnvc, primary neurovascular canal; rtg, replacement tooth germ; rtr, resorbed tooth roots; tb, trabecular bone. Scale bar is 20mm for a and 5mm for b, c, d, e.

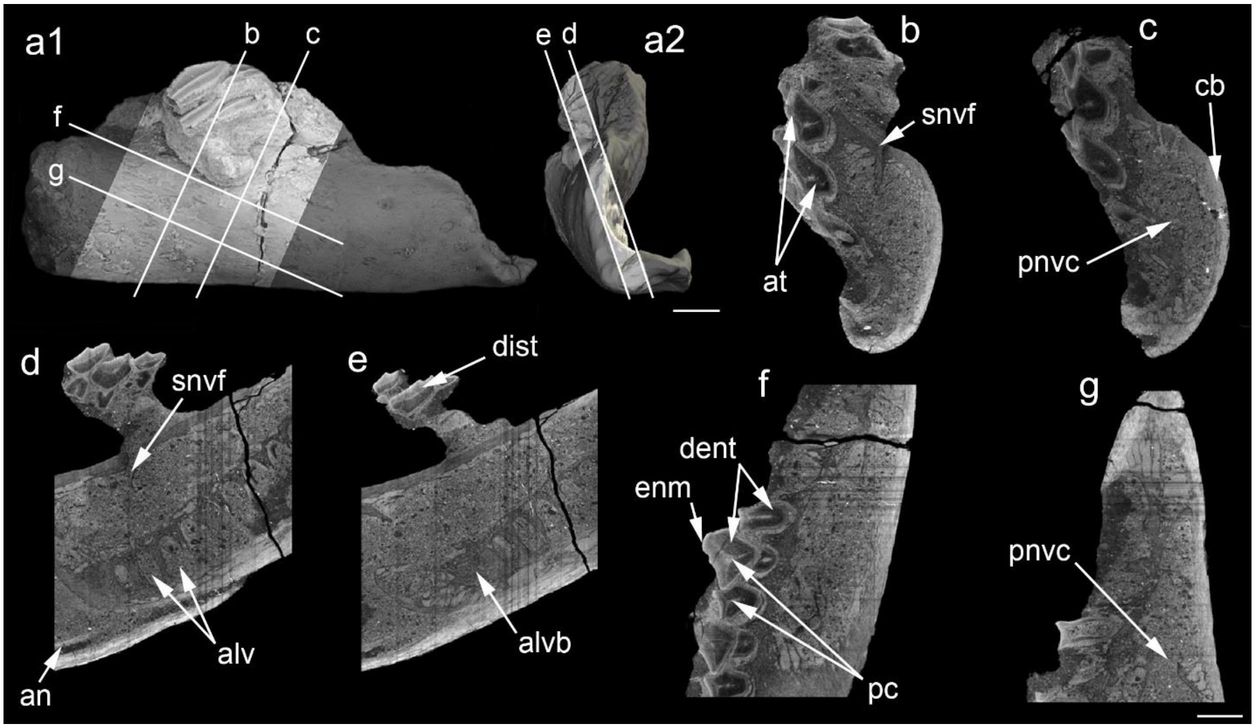

Supplementary Figure 10. Dentary of *Telmatosaurus transsylvanicus* (LPB [FGGUB] R.1305), normal right ramus, diagnostic micro-tomographic slices in the axial (b, c), sagittal (d, e) and coronal (f, g) planes. a1, general view illustrating the scan area and the range of slices selected in lateral view and **a2** in rostral view. (**b**) micro–CT image of axial slice no. 0402. (**c**) micro–CT image of axial slice no. 0791. (**d**) micro–CT image of sagittal slice no. 1058. (**e**) micro–CT image of sagittal slice no. 1122. (**f**) micro–CT image of coronal slice no. 0946. (**g**) micro–CT image of coronal slice no. 1349. Abbreviations: an, angular; alv, dentary alveoli; alvb, alveolar bone; at, active teeth; cb, cortical bone; dent, dentine; dist, displaced teeth; en, enamel; pc, pulp cavity; pnvc, primary neurovascular canal; snvf, secondary neurovascular foramen; Scale bar is 10mm for a1 and a2 and 5mm for b, c, d, e, f, g.

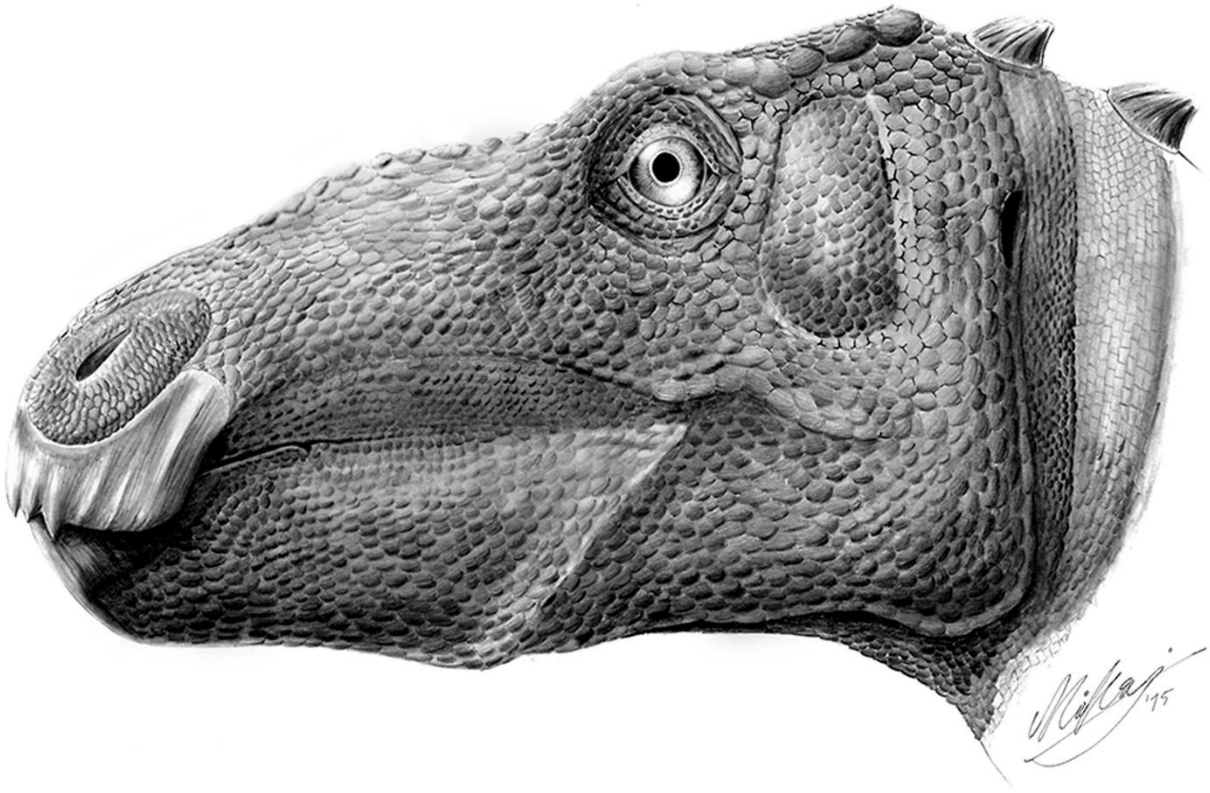

Supplementary Figure 11. Artistic reconstruction of the pathological individual hadrosauroid *Telmatosaurus transsylvanicus* in left lateral view showing the probable life appearance of the mandibular deformity. Reconstruction by M. D. D.

Supplementary Table 1. Differential diagnosis for ameloblastoma[1-9] in LPB (FGGUB) R.1305, pathologic left dentary of the hadrosauroid *Telmatosaurus transsylvanicus* (Nopcsa, 1900).

Pathology	Character	Density	Margin Definition	Margin border	Margin surface	Cortical expansion	Tooth roots	Trabeculae
Telmatosaurus dentary	Multilocular	Lytic & focal nodular	Well Defined	Opaque and pressure	Smooth	Present	Resorbed	None seen
**Ameloblastoma**	**Multilocular**	**Lytic & focal nodular**	**Well Defined**	**Opaque and pressure**	**Smooth**	**Present**	**Resorbed**	**Contains tooth**
Apical abscess/ granuloma	Unilocular or Multiloculate	Lytic	Well or ill defined	Normal bone	Irregular with sinus	Present	Resorbed	None seen
Osteomyelitis	Non-focal bone loss	Lytic or Opaque	Ill defined or Indistinct	Normal bone	Breakthrough	Present & periostitis	Resorbed	None seen
Tuberculosis	Unilocular or Multiple	Lytic or Opaque	Scalloped	Occasionall y sclerotic	Breakthrough	Present	Displaced	None seen
Keratocystic	Multiple or multilocular	Lytic	Well defined	Thin coalesced or scalloped	Smooth	Erosion & thinning	Resorbed & Displaced	Contains tooth
Radicular cyst (Periodontal cyst)	Unilocular	Lytic	Well or Ill defined	Opaque, undulating	Smooth	Present & thinning	Displaced	Contains tooth root
Ameloblastic fibroma	Multiloculated	Lytic	Well Defined	Pressure	Smooth	Present	Resorbed	Contains tooth
Odontogenic myxoma	Unilocular	Lytic	Ill defined	Normal bone	Smooth	Present & thinning	Resorbed	Septate Honeycomb
Primordial cyst	Unilocular	Lytic	Well Defined	Normal bone	Smooth	None	Displaced	Contains tooth
Odontogenic fibroma	Unilocular or multilocular	Lytic or Mixed	Well Defined	Normal bone	Smooth	None	Displaced	None seen
Traumatic bone cyst	Unilocular or multiple	Lytic	Well Defined	Opaque	Smooth or irregular	None	Absent	None seen
Solitary bone cyst (unicameral cyst)	Unilocular or Multilocular	Lytic	Well Defined	Opaque and scalloped	Smooth	Present & thinning	Absent	None seen
Aneurysmal bone cyst	Multilocular	Lytic	Well Defined	Opaque rim	Breakthrough	Present	Absent	Septae
Hemangioma	Unilocular	Lytic	Ill defined	Normal bone	Breakthrough	Slight & thinning	Resorbed	Thick trabeculae
Vascular malformation	Unilocular or multiple or multilocular	Lytic	Well Defined	Normal bone	Smooth	Present	Displaced	None seen
Intraosseous hematoma	Unilocular or multilocular	Lytic	Ill defined	Normal bone	Smooth	Absent	Resorbed	? Coarse
Odontoma	Unilocular or multilocular	Opaque	Well Defined	Normal bone	Pressure	Present & thinning	None	None seen
Enameloma	Unilocular	Opaque	Well defined	Normal bone	Smoth	Absent	None	None seen

Periapical cement dysplasia	U	Opaque	Well Defined	Round or lobulated	Marginal lucency	Absent	None	None seen
Eosinophilic granuloma	U or M	Lytic & opaque	Well or ill Defined	Normal bone	Lobulated or geographic	Present & periostitis	Floating	None seen
Ossified fibroma	Unilocular or multilocular	Lytic	Well Defined	Opaque Linear border	Smooth	Present & thinned	Displace	Ground glass
Osteoma	Unilocular	Lytic	Well Defined	Opaque	Smooth	Absent	Absent	None seen
Renal osteodystrophy	Uniolocular or Multilocular	Opaque	Well Defined	Normal bone	Smooth	Present & thinning	Displaced	Ground glass
Hyperparathyroid ism	Unilocular or multiple	Lytic	Well Defined	Normal bone	Scalloped	Present	Resorbed	Septae
Paget's disease	Unilocular	Lytic or opaque	Well or ill Defined	Normal bone	Indistinct or V-shaped	Present	Displaced Hyper-cementosi s	Ground glass & Thin
Fibrous dysplasia	Multilocular	Lytic	Ill Defined	Normal bone	Scalloped	Present & thinning	Resorbed	Ground glass
Giant cell tumor	Unilocular	Lytic	Well or ill Defined	Lobulated	Breakthrough	Present & thinnng	Displaced	Septae
Giant cell reparative granuloma	Unilocular or Multifocal or multilocular	Lytic	Well Defined	Normal bone	Smooth	Present	Displaced or Resorbed	None seen
Central giant cell granuloma	Multifocal	Lytic	Well Defined	Erosion	Breakthrough	Absent	Resorbed/ Displaced	Occasional trabeculae
Intraosseous neurilemmoma	Unifocal	Lytic	Well Defined	Normal bone	Breakthrough	Present	Displaced	None seen
Fibrosarcoma	Unilocular	Lytic	Ill Defined	Normal bone	Breakthrough	Present & thinning	Displaced	Septae
Osteosarcoma	Unilocular	Opaque or Lytic	Ill Defined	Normal bone	Breakthrough	Present & thinning	Displaced	Internal calcification
Mucoepidermoid carcinoma	Multilobular	Lytic	Ill Defined	Normal bone	Breakthrough	Present	Resorbed	Residual trabeculae
Multiple myeloma	Unilocular or multiple	Lytic or Opaque	Sharp or punched out	Normal bone	Smooth	Absent	Resorbed	None seen
Metastatic carcinoma	Unilocular or muiltiple	Lytic or Opaque	Ill Defined	Normal bone	Rough	Present	Resorbed or Displaced	None seen

Text S1.

Geological Setting and Taphonomic Context. The pathological specimen discussed in this report, LPB (FGGUB) R.1305 was discovered in an exposure of the Sînpetru Formation outcropping along the banks of the Sibişel River, in the central part of the Haţeg Basin (Fig. S2, S3a).

The Haţeg Basin is a small post-orogenic tectonic depression situated in the western part of the Southern Carpathians, part of the Cretaceous–Cenozoic Alps–Carpathians–Balkans orogenic chain. It formed in the aftermath of the Latest Cretaceous (Campanian–?early Maastrichtian) thrusting events (the Laramidian or Late Getidic orogenic phase[10]) responsible for the structuring of the Southern Carpathian segment of this late Alpine fold-and-thrust belt. The basin preserves a thick pile of Permian to Quaternary deposits[11] (Fig. S3a), separated into distinct sedimentary cycles by basin-scale unconformities.

The uppermost Cretaceous continental deposits, well-known for their vertebrate remains[12], form the basal-most part of the basin infill proper and are represented mainly by alternating sandstones, silty mudstones and mudstones, with subordinate conglomerate bodies (Fig. S3b); locally, volcanoclastic material is intimately associated with the continental siliciclastics.

These uppermost Cretaceous continental deposits are traditionally divided into two largely synchronous lithostratigraphic units, the volcano-detritic Densuş-Ciula Formation in the northwestern, and the siliciclastic Sînpetru Formation in the central parts of the basin[13]. These were deposited in alluvial plains of braided and meandering rivers surrounded by wide floodplains with generally moderate to good drainage, although areas of impeded drainage were also present locally; paleosol horizons are rather common throughout the deposits. The Haţeg paleo-rivers drained the nearby uplifted mountain areas of the Southern Carpathians, including the metamorphic core complex of the Retezat Mountains to the south[14] (Fig. S2).

The Maastrichtian age of these continental beds is rather well constrained, as they follow unconformably on top of upper Campanian marine beds. The faunal list of the rich and diverse vertebrate fauna of the Maastrichtian continental beds from Haţeg, first described by Nopcsa[15], has been seriously augmented by discoveries in the last decades and summarized[13]. Most recently reviewed by Csiki-Sava et

al.[16] and Weishampel and Jianu[17], the fauna consists of fish, anuran and albanerpetontid lissamphibians, multituberculate mammals, squamates (lizards and snakes), diverse chelonians and crocodyliforms, pterosaurs, and several dinosaurs: rhabdodontid and hadrosauroid ornithopods, nodosaurid ankylosaurs, titanosaurian sauropods, and a large variety of theropods including dromaeosaurids and birds. This peculiar and highly endemic fauna with dwarfed dinosaurs inhabited a subtropical island of the Late Cretaceous European Archipelago, located within the northern part of the Mediterranean Neo-Tethys[16]. Indeed, many of the Maastrichtian vertebrates from Haţeg (and more widely speaking, the Transylvanian region) are endemic, being restricted to this area. First of these taxa to be described was the 'duck-billed' hadrosaur (now regarded as a derived non-hadrosaurid hadrosauroid[18]) *Telmatosaurus transsylvanicus* (Nopcsa, 1900), whose remains are common, although not abundant, in the Maastrichtian of the Haţeg Basin; as we argue below, LPB (FGGUB) R.1305 can be referred to this taxon.

The Sibişel Valley outcrops were among the first deposits recognized as vertebrate-bearing continental beds in the Haţeg Basin[19]. Although their age was disputed at first - the fossiliferous Sînpetru 'sandstones' were assigned either to the lowermost Miocene or to the Cretaceous - they were definitively established as of Late Cretaceous age with the positive identification of dinosaur remains among the recovered vertebrate fossils. These outcrops are richly fossiliferous, and they yielded the major part of the material described by Nopcsa, including a partial skull with associated mandibles of *Telmatosaurus transsylvanicus* (Nopcsa, 1900), later selected as the lectotype of this taxon by Weishampel et al.[20].

The Sibişel Valley deposits represent the (as yet informal) stratotype of the Sînpetru Formation[13], and include repetitive fining-upward cycles of siliciclastic deposits ranging from conglomerates to sandstones, silty mudstones and mudstones with common and genetically diverse paleosol levels. Sedimentological studies of these deposits, exposed most optimally on the left side of the valley[14] (Fig. S2c and S3b) suggest that most of these were formed in a braided alluvial environment showing a complex and shifting mosaic of active channels, temporary wetlands and well-drained floodplain areas undergoing pedogenesis. Towards the upper part of the section, coarser grained beds (including coarse

conglomerates) and greyish mudstones dominate, suggesting tectonic and/or climatic activation of the source area (the paleo-Retezat Mountains) concomitant with the expansion of widespread wetlands[14].

LPB (FGGUB) R.1305 was collected in 1994 by students of the FGGUB in the lower-to-middle part of the left slope succession of the Sibișel section, in the upper part of the '6'-'7' composite section logged by Therrien[14] (Fig. S3). It was discovered in a bed of light grey to greenish fine sandstone, close to its slightly irregular base, lying approximately parallel to the general bedding plane of the deposits. The fossil-bearing bed can be interpreted as a crevasse deposits following Therrien[14]. Although the specimen was removed by students, its original setting was retraced shortly afterwards by one of the authors (Z. Cs.-S.), and the surrounding sediments were carefully screened for other vertebrate remains, but none were found in the proximity of the already excavated fossils.

The two rami of the lower jaw were preserved close to their *in vivo* articulation, with the symphyseal ends of the dentaries lying close to one another, while the distal ends were splayed. The two dentaries were, however, rotated inward along their longitudinal axes so that the medial sides of both rami were facing medioventrally, and the apical parts of the dental batteries were almost touching each other. Teeth are preserved partly *in situ* within both dentaries, arranged into the typical vertical rows of the apomorphic hadrosauroid dental battery, or else are slightly displaced from their original position while still retaining the reticulate disposition that characterizes the structure of the dental battery. The teeth closest to the rostral end of the right ramus have been displaced from their alveoli and rotated over the remaining teeth (Fig. S5d, S10e and 3D Fig. S5).

The preservation mode of the reddish dark brown-colored specimens is particularly good, with the cortical layer present and smooth, and no obvious signs of subaerial weathering or abrasion (3D Fig. S3). They show breakage at their distal ends, however, the postdentary elements of the lower jaws missing almost completely; based on its characteristics this breakage might be post-diagenetic, caused by recent erosion that exposed the dentaries shortly before their discovery. The teeth themselves are remarkably still arranged into dental batteries, and closely associated with their corresponding loci in the dentary (Movies S1 and S2 and 3D Figs S4, 5), whereas occurrence of toothless dentaries and isolated teeth

(fallen out shortly after death) appears to be the rule in the Haţeg fossil record of hadrosauroids (Z. Cs.-S., M. D. D., D. B. W., V. A. C., pers. observation), as seen in the lectotype dentaries of *Telmatosaurus* [Natural History Museum, United Kingdom (NHMUK) R.3386 – Suppl. Fig. S6]. The closely clustered arrangement of the two paired dentaries in the fossil-bearing bed, reminiscent of their natural articulated position, their commensurate dimensions and similar taphonomic features, as well as lack of any other vertebrate remains in their vicinity all strongly suggest that they represent elements derived from the lower jaw of a single individual.

Based on its taphonomic characteristics, it appears that although LPB (FGGUB) R.1305 obviously underwent advanced skeletal disarticulation, as well as some degree of fluvial transport and sorting, such processes were either not very intense and/or affected a fragmentary skull still showing anatomical connection of the bones, probably helped by retention of ligamentary connections or of some soft tissue covering. The sedimentary and taphonomic setting of this specimen conforms to the taphonomic mode "partial skeleton preserved in channel deposits" defined by Csiki et al.[21], and suggests burial of a skull fragment under relatively low-energy conditions during crevasse splay formation. Subsequent to burial, the sedimentary load pressed and rotated medially the lower jaw rami while also displacing slightly the dental batteries (especially that of the right dentary) and severing the interdentary connections in the symphyseal region.

Description and Taxonomic Identity of LPB (FGGUB) R.1305. The description of LPB (FGGUB) R.1305 is based largely on the left dentary that is both the pathological element under scrutiny, and the better preserved, more complete one, with completions and comparisons from its right counterpart whenever necessary.

The dentary is a horizontally elongated, roughly rectangular element with parallel and straight ventral and dorsal margins (Figs S4 and S5). It is obviously the largest bone of the mandible and contacted the predentary (not preserved) mesially, the angular (partially preserved only in the right ramus) (Fig. S10d; 3D Fig. S5) distoventrally, the splenial (not preserved) distomedially, and the surangular (not preserved)

distally. Between the extremely short diastema and the coronoid process, the lateral face of the dentary is strongly convex dorsoventrally for two thirds of its height and abruptly concave for the remaining upper third, forming the buccal recess that characterizes the tooth-bearing elements of most derived ornithopods[22-23].

The lateral surface of the dentary is slightly sinusoidal rostrocaudally in dorsal view (Figs S4d and S5d). Several neurovascular foramina are present in the upper third of the lateral surface, and another larger, primary foramen is placed lower, close to the articular surface for the predentary (Figs S4a and S5a).

The medial surface of the dentary is convex along both its rostrocaudal and dorsoventral axes, following the curvature of the dental battery that occupies most of the medial surface and extends behind the anterior margin of the coronoid process (Figs S4b and S5b). At the base of the dental battery, a sharp, slightly ventromedially-trending ridge separates the dental battery and the subdental alveolar foramina from the ventral border (Figs S4b and S5b).

The alveolar 'foramina' are arranged in a longitudinal row (Figs S4b and S5b). All foramina are similar in size and roughly elliptical in shape, with their long axes aligned antero-posteriorly. There are twelve foramina in the sequence, one foramen for each of the vertical alveoli surrounding the teeth. The condition of these structures as foramina representing possible transmission points for nervous or vascular structures is not consistent with the observations derived from the micro-tomographic slices as these do not communicate with the primary neurovascular canal (Movies S1 and S2), contra[24], and indeed are superficial in depth.

Ventral to the ventromedially oriented ridge lying below the row of foramina there is a deep mandibular groove (Meckelian groove) that runs rostrodistally, and is triangular in cross-section (Figs S4b and S5b); in life, it was covered by the splenial. The predentary articulation is convex rostromedially, and its articular surface is covered with numerous small ridges on its lower half (Figs S4a and S5a). The diastema is very short and thin mediolaterally; its dorsal margin is sharp and straight (Figs S4d and S5d). The rostral part of the dentary is strongly convex mediolaterally and the medial surface is deeply concave,

with its medioventral corner ventromedially beveled. The portion forming the dentary symphysis is slightly sinusoidal in outline (Figs S4c-d, S5c-d). The symphyseal surface has weakly overlapping bony prominences and a small, broad notch about two thirds from the rostral margin into which a corresponding bony spur of the right mandible interlocks (Figs S4b-c, S5b-c). The ventromedial part of the rostral dentary segment also shows a large semicircular foramen on its ventral surface, representing the opening for the large primary neurovascular canal (Figs S4c and S5c). Horizontal, rostrocaudal striations cover the surface anterior to this foramen.

The coronoid process projects laterodorsally from the lateral surface of the dentary, starting at a point that is anterior to the distal limit of the dental battery; it rises subvertically and projects markedly laterally relative to the tooth row, as is usual in the more derived iguanodontians (i.e., hadrosauroids[23]; Figs S4a-b). It is rostro-distally constricted at approximate mid-height; its terminal portion is not preserved (Fig. S4a). Below the coronoid process, a large mandibular (= Meckelian) fossa excavates the ventral part of the dentary (Fig. S4c).

The dental battery occupies most of the medial side of the dentary (Figs. S4b, S5b and 3D figures S4 and S5). Part of the alveolar parapet is preserved in the right ramus, covering the base of some of the teeth towards the rostral end of the element (Fig. S5b). The lateral edge of the dental battery is straight along its rostro-distal axis and the medial surface is convex along both its rostro-distal and dorso-ventral axes. The erupted dental battery is two teeth deep (3D Figs S4 and S5). The size of the individual teeth is proportionally larger compared to the dentary than in derived hadrosaurids due probably in part to its early ontogenetic developmental stage[25], but also to its taxonomic identity (see below).

Eight vertical tooth rows are preserved in the left dentary. The tooth rows are tightly arranged, partially overlapping one another; the functional teeth are identical in size (Figs S4b and S5b; 3D Figs S4 and S5). The continuous and dorsolabially-facing occlusal surface develops as a shallow depressed area across the apices of the functional tooth rows (Figs S4d and S5d). The height of the occlusal surface above the dorsal border of the dentary is lower in the pathologic left ramus (Fig. S4a and 3D Fig. S4) than in the normal right one (Fig. S5a and 3D Fig. S5). Moreover, in the left dentary, the tooth positions

opposite to the pathological portion are situated by 1.5mm below the level of the more distal and rostral tooth positions facing the dentary segments not affected by pathology. It is not clear whether the visible occlusal surface is natural or the result of breakage that could have led to the loss of the apical portion of the teeth in that area but breakage seems to be the best explanation.

In the pathologic dentary, some of the tooth roots display various degrees of root resorption. Some teeth have lost the roots almost completely (Fig. S9e). Meanwhile the teeth in the normal right ramus lack any such abnormalities. Replacement teeth (Fig. S7e) and tooth germs (Fig. S9e) can be seen immediately below and medial to the active teeth (Fig. S7e).

The two dentaries from Sânpetru show diagnostic characters that allow their assignment to Hadrosauroidea, most importantly the presence of a dental battery formed of alternating and tightly packed vertical tooth rows, comprising of at least two functional and one replacement tooth[20]. This assignment is also supported by the presence of an upright and laterally flared coronoid process, as well as by the moderately elongated, diamond-shape of the teeth with a single prominent median carina. Currently, only one taxon of hadrosauroid dinosaur was named from the Haţeg Basin (and the wider Transylvanian region) – *Telmatosaurus transsylvanicus* (Nopcsa, 1900), and hadrosauroid cranial, dental, and postcranial material discovered in this area is customarily referred to this taxon. However, Dalla Vecchia[26] has questioned this practice, and cautioned that the wide geographic and chronostratigraphic distribution of this material suggests the presence of more than one taxon. Regardless of such uncertainties, we feel secure in referring LPB (FGGUB) R.1305 to *Telmatosaurus transsylvanicus* because:

(1) There is overlap between our specimen and the lectotype of *Telmatosaurus* designated formally by Weishampel et al.[20] following Nopcsa's[27] description of the skull (without officially designating it as the holotype) a lectotype that includes both dentaries (NHMUK R.3386). Although the lectotype dentary is about 2.5 times larger than LPB (FGGUB) R.1305, and is devoid of teeth, it shares several features with the latter such as the relatively short, blocky and rectangular shape of the dentary body, subparallel dorsal and ventral margins, and the quasi-absence of a diastema between the predentary and the first tooth

position (Fig. S6). The main differences concern the number of tooth positions (26 in the lectotype – but this character is known to vary ontogenetically in hadrosauroids, increasing with age, and thus dentary size[23,25]), and the shape of the symphyseal area, which is somewhat ventrally projected and more ventro-rostrally inclined. However, the symphyseal area is broken and slightly displaced ventrally in the lectotype, so this feature might be exaggerated.

(2) The dentition preserved in LPB (FGGUB) R.1305 matches that described for *Telmatosaurus*, and shows several of the distinctive characters of this taxon, such as the relatively low number of successive replacement teeth in each tooth position as well as the autapomorphically relatively wide dentary teeth with a low and slightly mesially displaced median ridge, a crown with markedly denticulate margin, and mesial denticles supported by faint ridges[20].

(3) LPB (FGGUB) R.1305 comes not only from the same general area and succession as did the lectotype of *Telmatosaurus transsylvanicus*, but arguably from the very same part of the local succession. Although there is no clearly recorded position of the discovery site of NHMUK R.3386, according to the information that can be derived from Nopcsa's publication[27] this specimen came from the middle section of the left slope succession of the 'Sânpetru sandstones' along the Sibișel Valley, roughly at the same stratigraphic position as LPB (FGGUB) R.1305 (see suppl. ref. 21: fig. 1C – 'no. 16' marks the approximate position of the type locality of *Telmatosaurus*). The fact that the lectotype was discovered in a 'fossil pocket' – a typical bonebed-type accumulation that appears restricted to the lower-middle part of the Sibișel Valley section, but missing from its upper part[28] – also supports this assessment.

To conclude, the associated dentaries LPB (FGGUB) R.1305 can be thus reliably referred to *Telmatosaurus transsylvanicus* (Nopcsa, 1900), an autapomorphically dwarfed hadrosauroid dinosaur as documented by its osteohistologically proved small adult body size[29].

Internal Structure of the Dentary. The investigated portions of the rami are typified by an external layer of dense cortical bone (Figs S7d, S8c, S9d, S9c, S10c and Movies S1 and S2) and an internal layer of trabecular bone (Figs S7d, S8c, S9c and Movies S1 and S2).

The zone containing the pathological outgrowth has a severely modified cortical structure with a lytic density appearance (Figs S7e, S8e, S9e; Movies S1 and S2; 3D Fig. S4). The lytic appearance of the cortical bone is most conspicuous towards the anteroventral part of the outgrowth (3D Fig. S4). Cortical bone thinning is seen in several areas (Figs S7d, S8c and S9d).

Cortical bone expansion can be seen most clearly in coronal (Fig. S7c) and sagittal (Fig. S8e) views. This situation is in stark contrast to the condition seen in the normal right ramus whose cortical bone thickness and density remain constant throughout the investigated area without any of the abnormalities documented above.

Trabecular bone dominates the body of the elements, becoming slightly less dense in the alveolar bone (Figs S9d and S10e). The large primary neurovascular canal is traceable through the entire length of the investigated portion of the dentary (Movies S1 and S2; 3D Figs S4 and S5). It begins with an ovoid foramen that opens through the mandibular fossa and continues rostrally, lateral to the base of the dental battery and along the body of the dentary, finally exiting through the large mental foramen on the ventral surface of the edentulous rostral part (Figs S4c and S5c). Smaller, secondary neurovascular pathways branch rostrodorsally (Fig. S10b), rostrolaterally (Fig. S7c) or rostroventrally (S5c) from the primary neurovascular canal, and exit on the lateral wall of the dentary (3D Fig. S4).

The individual alveoli are not separated completely by alveolar bone walls. Each individual alveolus is pinched by the alveolar wall resulting in a “string-bead” appearance in sagittal view (Fig. S8*b* and 3D figures S4 and S5).

The *in-vivo* appearance of the pathological outgrowth of the left lower jaw (Fig. S11) would have closely followed the shape of deformation seen in the fossil specimen though somewhat enhanced by the soft tissue covering. This pathological condition was obviously not immediately life-threatening to the animal, as tooth growth was neither interrupted, nor seriously affected, and mandibular function appears to not have been hindered. However, the animal would have clearly been set apart by its facial malformation from other members of the herd. Whether the untimely death of this juvenile individual was a by-product of his benign mandibular neoplasia is unclear, but is certainly possible.

Supplementary References

1 Cankurtaran, C. Z., Branstetter, B. F., Chiosea, S. I. & Barnes, E. L. Ameloblastoma and Dentigerous Cyst Associated with Impacted Mandibular Third Molar Tooth. *RadioGraphics* **30**, 1415-1420 (2010).

2 Devenney-Cakir, B. *et al.* Cystic and Cystic-Appearing Lesions of the Mandible: Review. *Amer. J. Roentgenol.* **196**, 66-67 (2011).

3 Dunfee, B. L., Sakai, O., Pistey, R. & Gohel, A. Radiologic and pathologic characteristics of benign and malignant lesions of the mandible. *Radiographics* **26**, 1751-1768 (2006).

4 Jahanshahi, G., Haghighat, A. & Azmoodeh, F. Intraosseus neurilemmoma of the mandible: report of a rare ancient type. *Dental Res. J.* **8**, 150-153 (2011).

5 Kawai, N., Wakasa, T., Asaumi, J-I. & Kishji, K. A radiographic study on resorption of tooth root associated with malignant tumors. *Oral Radiol.* **16**, 55-65 (2000).

6 Meyer, K. A., Bancroft, L. W., Dietrich, T. J., Kransdorf, M. J. & Peterson, J. J. Imaging characteristics of Benign, Malignant and Infectious Jaw Lesions: A Pictoral Review. *Amer. J. Roentgenol.* **197**, 412-421 (2011).

7 Resnick, D. *Diagnosis of Bone and Joint Disorders*. **4**. (Philadelphia, Saunders, 2002).

8 Simon, D., Somanathan, T., Ramdas, K. & Pandey, M. Central Mucoepidermoid carcinoma of mandible - A case report and review of the literature. *World J. Surg. Oncol.* **1**, 1-6 (2003).

9 Wuehrmann, A. H. & Manson-Hing, L. R. *Dental Radiology* (St. Louis, Mosby, 1973).

10 Săndulescu, M. *Geotectonica României* (Bucharest, Editura Tehnică, 1984).

11 Stilla, A. Geologie de la region de Haţeg-Cioclovina-Pui-Băniţa (Carpathes Meridionales*). Anuarul Instit. Geol. Geofiz.* **66**, 92-197 (1985).

12 Grigorescu, D. The Latest Cretaceous fauna with dinosaurs and mammals from the Haţeg Basin - A historial overview. *Palaeogeogr. Palaeoclimatol. Palaeoecol*. **293**, 271-282 (2010).

13 Grigorescu, D. in *Aspects of Nonmarine Cretaceous Geology*. 142-164 (Beijing, China Ocean Press, 1992).

14 Therrien, F. Depositional environments and fluvial system changes in the dinosaur-bearing Sânpetru Formation (Late Cretaceous, Romania): post-orogenic sedimentation in an active extensional basin. *Sedim. Geol.* **192**, 183-205 (2006).

15 Nopcsa, F. Die Dinosaurier der Siebenbuergischen Landesteile Ungarns. *Mitt. Jahr. Tc. -ung. geol. Reichsanstalt* **23**, 1-24 (1915).

16 Csiki-Sava, Z., Buffetaut, E., Ösi, A., Pereda-Superbiola, X. & Brusatte, S. Island life in the Cretaceous-faunal compositon, biogeography, evolution, and extinction of land-living vertebrates of the Late Cretaceous European archipelego. *Zookeys* **469**, 1-161 (2015).

17 Weishampel, D. B. & Jianu, C. M. *Transylvanian Dinosaurs* (Baltimore, The John Hopkins University Press, 2011).

18 Prieto-Marquez, A. Global phylogeny of Hadrosauridae (Dinosauria: Ornithopoda) using parsimony and Bayesian methods. *Zool. J. Linn. Soc.* **159**, 435-502 (2010).

19 Halaváts, G. Y. Adatok a Hátszegi medence földtani viszonyainak ismeretéhez. *M. kir. Földt. Intézet, Évi Jel.* **1896-ról**, 90-95 (1897).

20 Weishampel, D. B., Norman, D. B. & Grigorescu, D. *Telmatosaurus transsylvanicus* from the Late Cretaceous of Romania: the most basal hadrosaurid dinosaur. *Palaeontology* **36**, 361-386 (1993).

21 Csiki, Z., Grigorescu, D., Codrea, V. & Therrien, F. Taphonomic modes in the Maastrichtian continental deposits of the Haţeg Basin, Romania - Palaeoecological and palaeobiological inferences. *Palaeogeogr. Palaeoclimatol. Palaeoecol.* **293**, 375-390 (2010).

22 Norman, D. B., Sues, H-D., Witmer, L. M. & Coria, R. A. in *The Dinosauria*, *Second Edition*. 393-412 (Berkeley, University of California Press, 2004).

23 Norman, D. B. in *The Dinosauria*, *Second Edition*. 413-437 (Berkeley, University of California Press, 2004).

24 Holliday, C. M. New Insights Into Dinosaur Muscle Anatomy. *The Anat. Rec*. **292**, 1246-1265, (2009).

25 Weishampel, D. B. Evolution of Jaw Mechanisms in Ornithopod Dinosaurs. *Adv. Anat. Embryol. Cell Biol.* **87**, 1-58 (1984).

26 Dalla Vecchia, F. M. in *Hadrosaurs*. *Life of the Past.* 268-297 (Bloomington and Indianapolis, Indiana University Press, 2014).

27 Nopcsa, F. Dinosaurierreste aus Siebenbürgen I. Schädel von *Limnosaurus transsylvanicus* nov. gen.et nov. spec. *Denkschr. K. Akad. Wiss. Math.-naturwiss. Klasse* **68**, 555-591 (1900).

28 Therrien, F., Zelenitsky, D. K. & Weishampel, D. B. Palaeoenvironmental reconstruction of the Late Cretaceous Sânpetru Formation (Hațeg Basin, Romania) using paleosols and implications for the 'disappearance' of dinosaurs. *Palaeogeogr. Palaeoclimatol. Palaeoecol*. **272**, 37-52 (2009).

29 Benton, M. J. *et al.* Dinosaurs and the island rule: The dwarfed dinosaurs from Haţeg Island, *Palaeogeogr. Palaeoclimatol. Palaeoecol*. **293**, 438-454 (2010).

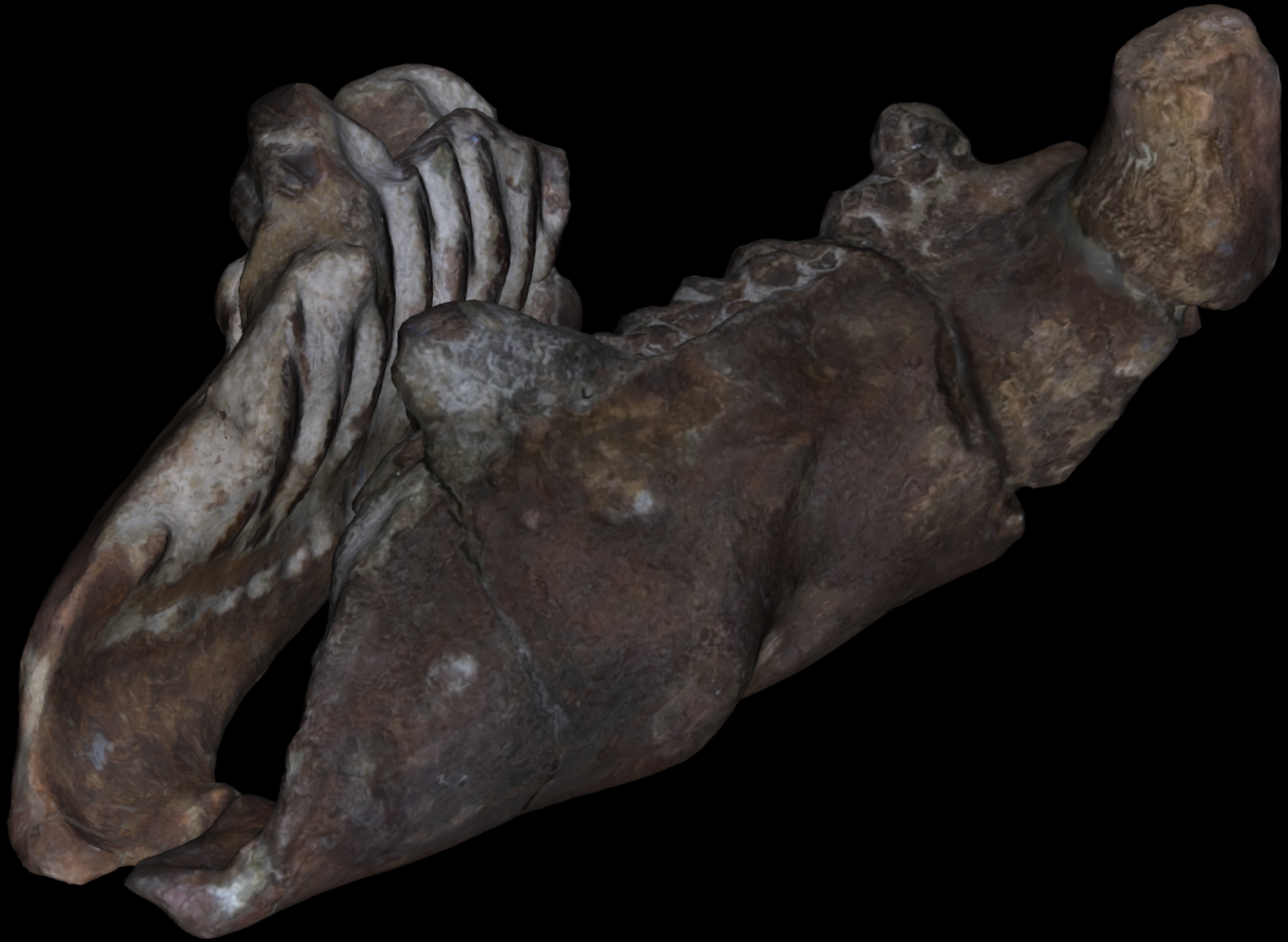

3D Figure S3

3D surface scan of the *Telmatosaurus transsylvanicus* LPB (FGGUB) R.1305, articulated mandibular rami.

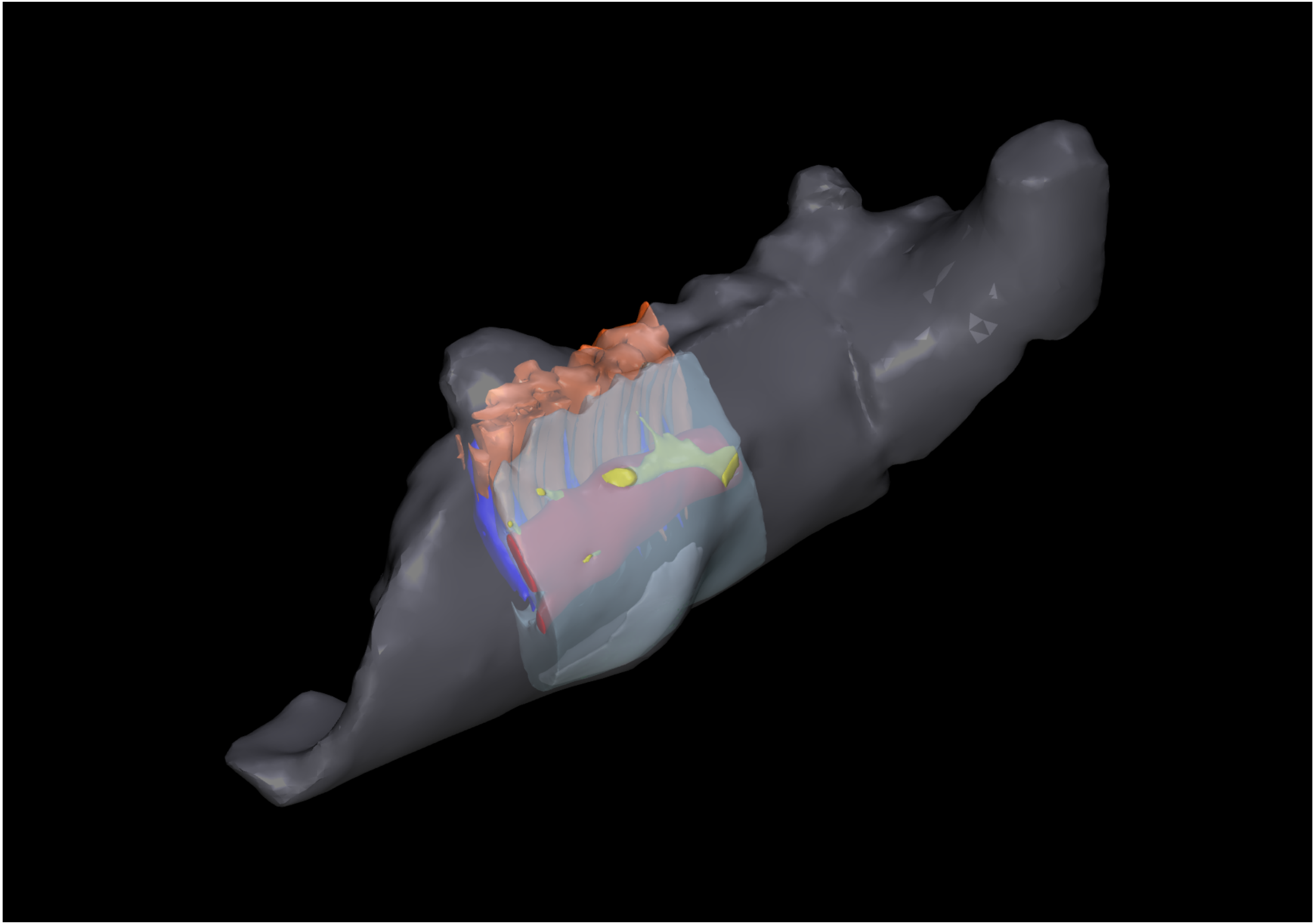

3D Figure S4

Telmatosaurus transsylvanicus LPB (FGGUB) R.1305, left mandibular ramus, segmented internal structures. Elements are color-coded as follows: external ramus surface, white; functional teeth, orange; replacement teeth, blue; dentary bone, light blue; primary neurovascular canal, red; secondary neurovascular network, yellow; internal extent of pathology, bright white; lytic density areas, deep green. The dentary and pathology have been made transparent in order to visualize the internal anatomy.

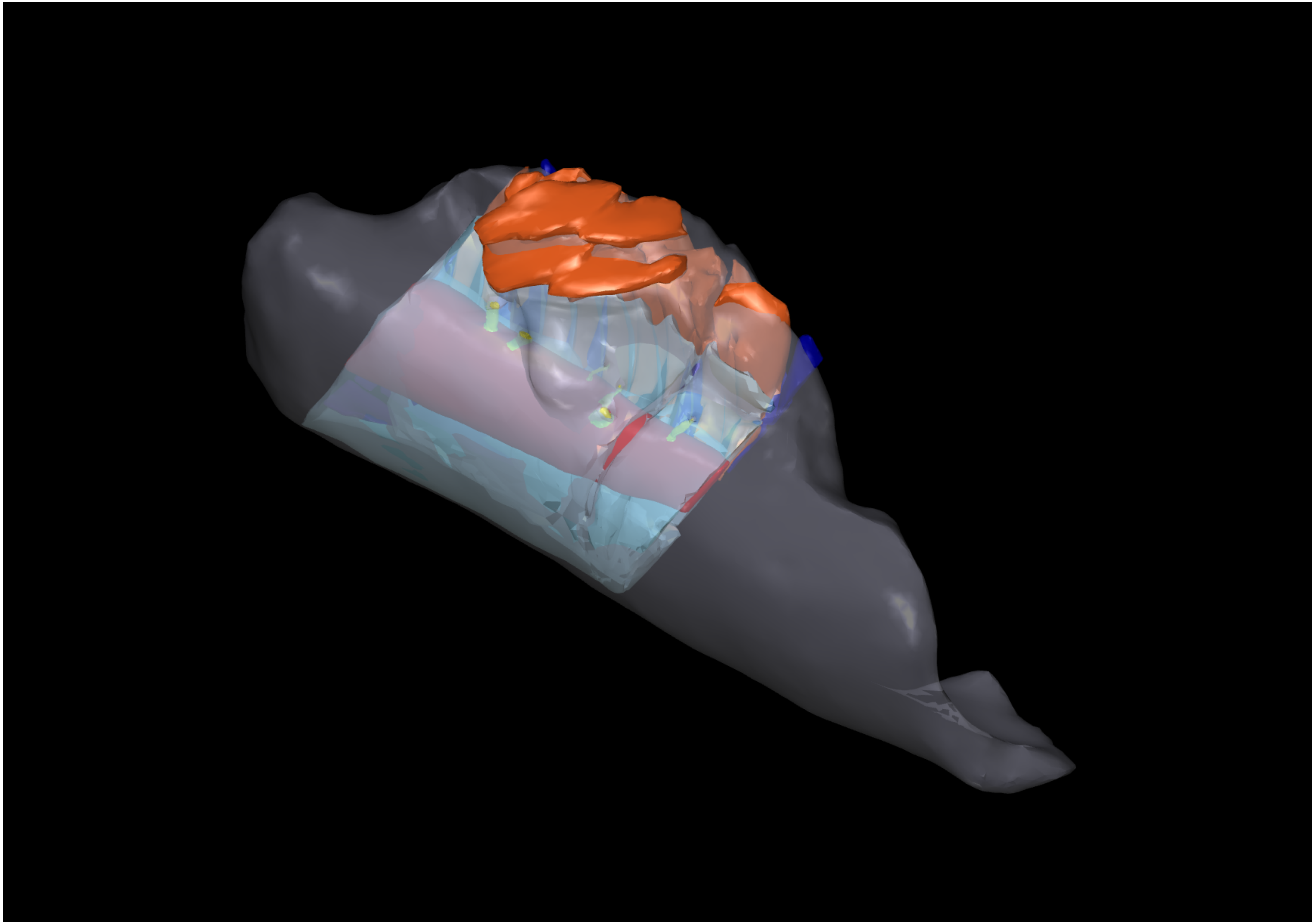

3D Figure S5

Telmatosaurus transsylvanicus LPB (FGGUB) R.1305, right mandibular ramus, segmented internal structures. Elements are color-coded as follows: external ramus surface, white; functional teeth, orange; replacement teeth, blue; dentary bone, light blue; primary neurovascular canal, red; secondary neurovascular network, yellow. The dentary has been made transparent in order to visualize the internal anatomy.
